# Active inference, eye movements and oculomotor delays

**DOI:** 10.1007/s00422-014-0620-8

**Published:** 2014-08-16

**Authors:** Laurent U. Perrinet, Rick A. Adams, Karl J. Friston

**Affiliations:** 1 Institut de Neurosciences de la Timone, CNRS/Aix-Marseille Université, Marseille, France; 2 The Wellcome Trust Centre for Neuroimaging, University College London, London, UK

**Keywords:** Oculomotor delays, Tracking eye movements, Smooth pursuit eye movements, Generalised coordinates, Variational free energy, Active inference

## Abstract

This paper considers the problem of sensorimotor delays in the optimal control of (smooth) eye movements under uncertainty. Specifically, we consider delays in the visuo-oculomotor loop and their implications for active inference. Active inference uses a generalisation of Kalman filtering to provide Bayes optimal estimates of hidden states and action in generalised coordinates of motion. Representing hidden states in generalised coordinates provides a simple way of compensating for both sensory and oculomotor delays. The efficacy of this scheme is illustrated using neuronal simulations of pursuit initiation responses, with and without compensation. We then consider an extension of the generative model to simulate smooth pursuit eye movements—in which the visuo-oculomotor system believes both the target and its centre of gaze are attracted to a (hidden) point moving in the visual field. Finally, the generative model is equipped with a hierarchical structure, so that it can recognise and remember unseen (occluded) trajectories and emit anticipatory responses. These simulations speak to a straightforward and neurobiologically plausible solution to the generic problem of integrating information from different sources with different temporal delays and the particular difficulties encountered when a system—like the oculomotor system—tries to control its environment with delayed signals.

## Introduction

### Problem statement

This paper considers optimal motor control and the particular problems caused by the inevitable delay between the emission of motor commands and their sensory consequences. This is a generic problem that we illustrate within the context of oculomotor control where it is particularly prescient (see for instance (Nijhawan 2008) for a review). Although we focus on oculomotor control, the more general contribution of this work is to treat motor control as a pure inference problem. This allows us to use standard (Bayesian filtering) schemes to resolve the problem of sensorimotor delays—by absorbing them into a generative or forward model. Furthermore, this principled and generic solution has some degree of biological plausibility because the resulting active (Bayesian) filtering is formally identical to predictive coding, which has become an established metaphor for neuronal message passing in the brain. We will use oculomotor control as a vehicle to illustrate the basic idea using a series of generative models of eye movements—that address increasingly complicated aspects of oculomotor control. In short, we offer a general solution to the problem of sensorimotor delays in motor control—using established models of message passing in the brain—and demonstrate the implications of this solution in the particular setting of oculomotor control.

**Fig. 1:**
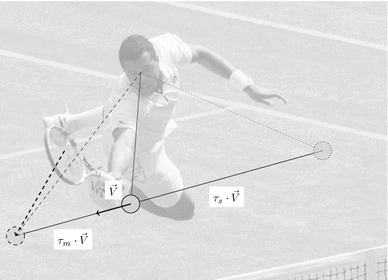
Problem statement: optimal motor control under axonal delays. The central nervous system has to contend with axonal delays, both at the sensory and the motor levels. For instance, in the human visuo-oculomotor system, it takes approximately $\tau _s=50~\hbox {ms}$ for the retinal image to reach the visual areas implicated in motion detection and a further $\tau _m=40~\hbox {ms}$ to reach the oculomotor muscles. As a consequence, for a tennis player trying to intercept a ball at a speed of $20~\hbox {m}\,\hbox {s}^{-1}$, the sensed physical position is $1~\hbox {m}$ behind the true position (as represented here by $\tau _s \cdot \varvec{V}$), while the position at the moment of emitting the motor command will be $.8~\hbox {m}$ ahead of its execution ($\tau _m \cdot \varvec{V}$). Note that while the actual position of the ball when its image produced by the photoreceptors on the retina hits visual areas is approximately at $45$ degrees of eccentricity (*red dotted line*), the player’s gaze is directed to the ball at its present position (*red line*), in anticipatory fashion. Optimal control directs action (future motion of the eye) to the expected position (*red dashed line*) of the ball in the future—and the racket (*black dashed line*) to the expected position of the ball when motor commands reach the periphery (muscles)

The oculomotor system produces eye movements to deploy sensory (retinal) epithelia at very fast timescales. In particular, changes in the position of a visual object are compensated for with robust and rapid eye movements, such that the object is perceived as invariant, despite its motion (Ilg 1997; Lisberger et al. 1987). This near-optimal control is remarkable, given the absence of any external clock to coordinate dynamics in different parts of the visual–oculomotor system. An important constraint, in this setting, is axonal conduction, which produces delays in sensory and motor signalling within the oculomotor system. Figure 1 shows that in humans, for example, retinal signals arriving at motion processing areas report the state of affairs at least about 50 ms ago, while the action that follows is executed at least 40 ms in the future (Inui and Kakigi 2006); for a review, see Masson and Ilg (2010). Different sources of delays exist—such as the biomechanical delay between neuromuscular excitation and eye movement. Due to these delays, the human smooth pursuit system responds to unpredictable stimuli with a minimum latency of around 100 ms (Wyatt and Pola 1987). In addition, these delays may produce oscillations about a constant velocity stimulus (Robinson et al. 1986; Robinson 1965), whose amplitude and frequency can be altered by artificially manipulating the feedback (Goldreich et al. 1992).

Eye movements can anticipate predictable stimuli, such as the sinusoidal movement of a pendulum (Barnes and Asselman 1991; Dodge et al. 1930; Westheimer 1954); for a review, see Barnes (2008). Interestingly, ocular tracking can compensate for sensorimotor delays after around one or two periods of sinusoidal motion—producing a tracking movement with little discernible delay (Barnes and Asselman 1991). This suggests that the oculomotor system can use sensory information from the past to predict its future sensory states (including its actions), despite the fact that these sensory changes can be due to both movement of the stimulus and movement of the eyes. The time taken to compensate for delays increases with the unpredictability of the stimulus (Michael and Jones 1966), though the system can adapt quickly to complex waveforms, with changes in velocity (Barnes and Schmid 2002), single cycles (Barnes et al. 2000) or perturbed periodic waves—where subjects appear to estimate their frequency using an average over recent cycles (Collins and Barnes 2009). Further studies suggest that different sources of information, such as auditory or verbal cues (Kowler 1989) or prior knowledge about the nature of sensory inputs (Montagnini et al. 2006), can evoke anticipatory eye movements.

The aim of this work was to establish a principled model of optimal visual motion processing and oculomotor control in the context of sensorimotor delays. Delays are often ignored in treatments of the visual–oculomotor system; however, they are crucial to understanding the system’s dynamics. For instance, delays may be important for understanding the pathophysiology of impaired oculomotor control: schizophrenic smooth pursuit abnormalities are due to impairments of the predictive (extra-retinal) motion signals that are required to compensate for sensorimotor delays (Nkam et al. 2010; Thaker et al. 1999). Surprisingly, delays may also explain a whole body of visual illusions (Changizi 2001; Changizi and Widders 2002; Changizi et al. 2008; Vaughn and Eagleman 2013), even for visual illusions that involve a static display. Delays are also an important consideration in control theory and engineering. Finally, neuronal solutions to the delay problem speak to the representation of time in the brain, which is essential for the proper fusion of information in the central nervous system.

### Existing solutions and the proposed hypothesis

A principled approach to optimal oculomotor control is provided by Bayesian filtering schemes that use probabilistic representations to estimate visual and oculomotor states. These states are *hidden*; i.e. they cannot be measured directly. A popular scheme for linear control problems is the Kalman filter (Kalman 1960). The Kalman scheme can be extended to accommodate biomechanical constraints, such as transmission delays (e.g. fixed-lag smoothers). However, their solutions can become computationally complex when delays are large in relation to discretisation time and are not biologically plausible. We have previously considered generalised Bayesian filtering in continuous time as a metaphor for action and perception. This approach has been applied to eye movements (Friston et al. 2010b) and saccades in particular (Friston et al. 2012a). However, these applications ignored sensorimotor delays and their potentially confounding effects on optimal control.

Crucially, the active inference schemes we have considered previously are formulated using representations in *generalised coordinates of motion*; that is, states (such as position) are represented along with their higher-order temporal derivatives (such as speed, acceleration and jerk). This means that one has an implicit representation of hidden states in the recent past and future that can be used to finesse the problems of delays. For example, it has been shown that acceleration is a necessary component of the predictive drive to eye movements (Bennett et al. 2007). In brief, generalised representations can be projected to the past and to the future using simple (linear) mixtures of generalised motion. Note that a representation of generalised motion can be explicit or implicit using a population coding scheme—as has been demonstrated for acceleration (Lisberger and Movshon 1999). Representations of generalised motion may be important for modelling delays when integrating information in the brain from distal sources—such as other cortical columns in the same cortical area or other areas that are connected with fixed but different delays (Roelfsema et al. 1997). The integration of information over time becomes particularly acute in motor control, where the products of sensory processing couple back to the sampling of sensory information through action.

In the context of action, acted inference finesses the problems with delayed control signals in classical formulations of motor control by replacing command signals with descending corticospinal predictions. For instance, the location of receptive fields in the parietal cortex in monkeys is shown to shift transiently before an eye movement (Duhamel et al. 1992). These predictions are fulfilled at the peripheral level, using fast closed loop mechanisms (peripheral reflex arcs). In principle, “these predictions can anticipate delays if they are part of the generative model,” (Friston 2011); however, this anticipation has never been demonstrated formally. Here, we show how generalised Bayesian filtering—as used in active inference—can compensate for both sensory and motor delays in the visual–oculomotor loop.

It is important to mention what this work does not address. First, we focus on tracking eye movements (pursuit of a single-dot stimulus for a monocular observer with fixed head position): we do not consider other types of eye movements (vergence, saccades or the vestibulo-ocular reflex). Second, we take an approach that complements existing models, such as those of Robinson et al. (1986) and Krauzlis and Lisberger (1989). Existing models account for neurophysiological and behavioural data by refining block diagram models of oculomotor control to describe *how* the system might work. We take a more generic approach, in which we define the imperatives for any system sampling sensory data, derive an optimal oculomotor control solution and show *why* this solution explains the data. Although the two approaches should be consistent, ours offers a principled approach to identifying the necessary solutions (such as predictive coding) to a given problem (oculomotor delays). We hope to demonstrate the approach by modelling pursuit initiation and smooth pursuit—and then consider the outstanding issue of anticipatory responses: in previous treatments (Robinson et al. 1986), “[anticipation] has not been adequately modelled and no such attempt is offered (...) only unpredictable movements are considered”.

### Outline

The main contributions of our work are described in the subsequent five sections. First, sect. 2 summarises the basic theory behind active inference and attempts to link generalised filtering to conventional Bayesian filters used in optimal control theory. This section then considers neurobiological implementations of generalised filtering, in terms of predictive coding in generalised coordinates of motion. This formulation allows us to consider the problem of delayed sensory input and motor output in sect. 3—and how this problem can be finessed in a relatively straightforward way using generalised representations. Having established the formal framework (and putative neuronal implementation), the final three sections deal with successively harder problems in oculomotor control. We start in Sect. 4 by considering *pursuit initiation* using a simple generative model of oculomotor trajectories. Using simulations, we consider the impact of motor delays, sensory delays and their interaction on responses to a single sweep of a visual target. The subsequent section turns to *smooth pursuit eye movements*—using a more sophisticated generative model of oculomotor trajectories, in which prior beliefs about eye movements enable the centre of gaze to predict target motion using a virtual or fictive target (see Sect. 5). In the final section, we turn to hierarchical models of target trajectories that have explicit memories of hidden dynamics, which enable anticipatory responses (see Sect. 6). These responses are illustrated using simulations of anticipatory pursuit movements using (rectified) hemi-sinusoidal motion. In short, these theoretical considerations lead to a partition of stimulus-bound eye movements into pursuit initiation, smooth pursuit and anticipatory pursuit, where each mode of oculomotor control calls on formal additions to the underlying generative model; however, they all use exactly the same scheme and basic principles. Where possible, we try to simulate classic empirical results in this field—at least heuristically.

In short, these theoretical considerations lead to a partition of stimulus-bound eye movements into pursuit initiation, smooth pursuit and anticipatory pursuit, where each mode of oculomotor control calls on formal additions to the underlying generative model. However, these models all use exactly the same scheme and basic principles; in particular, they all use the same solution to the oculomotor delay problem. These simulations illustrate that the active inference scheme can reproduce classical empirical results in three distinct experimental contexts.

## From predictive coding to active inference

This section sets out the basic theory, before applying it to the special problem of oculomotor delays in the following sections. We first introduce the general framework of active inference in terms of generalised Bayesian filtering and variational free energy minimisation. In brief, active inference can be regarded as equipping standard Bayesian filtering schemes with classical reflex arcs that enable action, such as an eye movement, to fulfil predictions about hidden states of the world. Second, we will briefly describe the formalism of active inference in terms of differential equations describing the dynamics of the world and internal states of the visual–oculomotor system. The neurobiological implementation of these differential equations is considered in terms of predictive coding, which uses prediction errors on the motion of hidden states—such as the location of a visual target. In the next section, we will turn to the special problem of oculomotor delays and how this problem can be finessed using active inference in generalised coordinates of motion. This solution will be illustrated in subsequent sections using simulations of pursuit initiation responses and smooth pursuit. Finally, we shall exploit the richness of hierarchical generative models—which underlie active inference—to illustrate anticipatory eye movements that cannot be explained by simply compensating for oculomotor delays.

### From free energy to generalised filtering

The scheme used here to model oculomotor behaviour has been used to model several other processes and paradigms in neuroscience. This active inference scheme is based upon three assumptions:

- The brain minimises the free energy of sensory inputs defined by a generative model.
- The generative model used by the brain is hierarchical, nonlinear and dynamic.
- Neuronal firing rates encode the expected state of the world, under this model.

The first assumption is the free energy principle, which leads to active inference in the context of an embodied interaction of the system with its environment, where the system can act to change its sensory inputs. The free energy here is a variational free energy that provides a computationally tractable upper bound on the negative logarithm of Bayesian model evidence (see Appendix 1). In Bayesian terms, this means that the brain maximises the evidence for its model of sensory inputs (Ballard et al. 1983; Bialek et al. 2001; Dayan et al. 1995; Gregory 1980; Grossberg et al. 1997; Knill and Pouget 2004; Olshausen and Field 1996). This is the Bayesian brain hypothesis (Yuille and Kersten 2006). If we also allow action to maximise model evidence, we get active inference (Friston et al. 2010b). Crucially, unlike conventional optimal control schemes, there is no *ad hoc* value or loss function guiding action: action minimises the free energy of the system’s model. This permits the application of standard Bayesian solutions and simplifies the implicit neuronal architecture; for example, there is no need for an efference copy signal (Friston 2011). In this setting, desired movements are specified in terms of prior beliefs about state transitions or the motion of hidden states in the generative model. Action then realises prior beliefs (policies) by sampling sensory data that provide evidence for those beliefs.

The second assumption above is motivated by noting that the world is both dynamic and nonlinear—and that hierarchical structure emerges inevitably from a separation of temporal scales (Ginzburg 1955; Haken 1983). The third assumption is the Laplace assumption that, in terms of neural codes, leads to the *Laplace code*, which is arguably the simplest and most flexible of all neural codes (Friston 2009). In brief, the Laplace code means that probabilistic representations are encoded explicitly by synaptic activity in terms of their mean or expectation (while the second-order statistics such as dispersion or precision are encoded implicitly in terms of synaptic activity and efficacy). This limits the representation of hidden states to continuous variables, as opposed to discrete states; however, this is appropriate for most aspects of sensorimotor processing. Furthermore, it finesses the combinatoric explosion associated with discrete state space models. Restricting probabilistic representations to a Gaussian form clearly precludes multimodal representations. Having said this, the hierarchical form of the generative models allows for fairly graceful modelling of nonlinear effects (such as shadows and occlusions). For example, a Gaussian variable at one level of the model may enter the lower levels in highly nonlinear way—we will see examples of this later. See Appendix 2 for a motivation of the Laplace assumption from basic principles.

Under these assumptions, action and perception can be regarded as the solutions to coupled differential equations describing the dynamics of the real world (the first pair of equations) and the behaviour of an agent (the second pair of equations), expressed in terms of action and internal states that encode conditional expectations about hidden states of the world (Friston et al. 2010b):

1 $$\begin{aligned}&\varvec{s} = \varvec{g(x, \nu , a) + {\omega _\nu }} \nonumber \\&\dot{\varvec{x}}= \varvec{f(x, \nu , a) + \omega _x} \nonumber \\&\dot{a} = -{\partial _a} F(\tilde{s}, \tilde{\mu }) \nonumber \\&{\dot{\tilde{\mu }}} = {\fancyscript{D}} {\tilde{\mu }} - {\partial _{{\tilde{\mu }}}} F({\tilde{s}}, {\tilde{\mu }}) \end{aligned}$$

For clarity, real-world states are written in boldface, while internal states of the agent are in italics: Here, $\varvec{(s, x, \nu , a)}$ denote sensory input, hidden states, hidden causes and action in the real world, respectively. The variables in the second pair of equations $(\tilde{s}, \tilde{\mu }, a)$ correspond to generalised sensory input, conditional expectations and action. Generalised coordinates of motion, denoted by the ~ notation, correspond to a vector representing the different orders of motion of a variable: position, velocity, acceleration and so on (Friston et al. 2010a). Using the Lagrangian notation for temporal derivatives, we get, e.g., for $s$: $\tilde{s} = (s, s^{\prime }, s^{\prime \prime }, \ldots )$. In the absence of delays $\tilde{s}(t) = \varvec{\tilde{s}}(t)$, the agent receives instantaneous sensations from the real world. The differential equations above are coupled because sensory states depend upon action through hidden states and causes $\varvec{(x, \nu )}$ while action $a(t) = \varvec{a}(t)$ depends upon sensory states through internal states $\tilde{\mu }$.

By explicitly separating real-world states—hidden from the agent—to its internal states, one can clearly separate the generative model from the updating scheme that allows to minimise the agent’s free energy: the first pair of coupled stochastic differential equations describes the dynamics of hidden states and causes in the world and how they generate sensory states. These equations are stochastic because sensory states and the motion of hidden states are subject to random fluctuations $(\varvec{\omega _x, \omega _\nu })$.

**Fig. 2:**
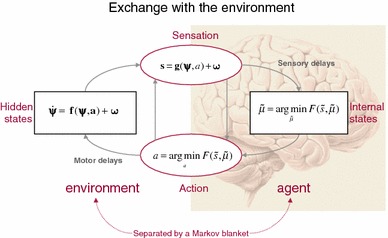
This *schematic* shows the dependencies among various quantities modelling exchanges of an agent with the environment. It shows the states of the environment and the system in terms of a probabilistic dependency graph, where connections denote directed dependencies. The quantities are described within the nodes of this graph—with exemplar forms for their dependencies on other variables (see main text). Hidden (external) and internal states of the agent are separated by action and sensory states. Both action and internal states—encoding a conditional probability density function over hidden states—minimise free energy. Note that hidden states in the real world and the form of their dynamics can be different from that assumed by the generative model; this is why hidden states are in *bold*. See main text for further details

The second pair of differential equations corresponds to action and perception, respectively—they constitute a (generalised) gradient descent on variational free energy. The differential equation describing changes in conditional expectations (perception) is known as *generalised filtering* or predictive coding and has the same form as standard Bayesian (Kalman–Bucy) filters—see also Beal (2003) and Rao and Ballard (1999). The first term is a prediction based upon a differential operator $\fancyscript{D}$ that returns the generalised motion of the conditional expectations, namely the vector of velocity, acceleration, jerk and so on—such that $\fancyscript{D}\tilde{\mu } = (\mu ^{\prime }, \mu ^{\prime \prime }, \mu ^{\prime \prime \prime }, \ldots )$. However, the expected velocity is not the velocity of the expectation and comprises both prediction and update terms: the second term reflects this correction and ensures the changes in conditional expectations are Bayes optimal predictions of hidden states of the world—in the sense that they maximise the free-energy bound on Bayesian model evidence. See Fig. 2 for a schematic summary of the implicit conditional dependencies implied by Eq. 1.

### Hierarchical form of the generative model

To perform simulations using this scheme, one simply integrates or solves Eq. 1 to simulate (neuronal) dynamics that encode conditional expectations and ensuing action. Conditional expectations depend upon a generative model, which we assume has the following (hierarchical) form

2 $$\begin{aligned} s&= g^{(1)}(x^{(1)}, v^{(1)}) + \omega ^{(1)}_\nu \nonumber \\ \dot{x}^{(1)}&= f^{(1)}(x^{(1)}, v^{(1)}) + \omega _x^{(1)} \nonumber \\&\vdots&\nonumber \\ \nu ^{(i-1)}&= g^{(i)}(x^{(i)}, v^{(i)}) + \omega ^{(i)}_\nu \nonumber \\ \dot{x}^{(i)}&= f^{(i)}(x^{(i)}, v^{(i)}) + \omega _x^{(i)}\nonumber \\&\vdots&\end{aligned}$$

where $(i)$ indexes the level in the hierarchical model. Note that we denote the sensory layer as $i=0$, but this indexing is somewhat arbitrary. This equation is just a way of writing down a generative model that specifies a probability density function over sensory inputs and hidden states and causes. This probability density is needed to define the free energy of sensory input: it is specified in terms of functions $(f^{(i)} , g^{(i)})$ and Gaussian assumptions about random fluctuations $(\omega ^{(i)}_x, \omega ^{(i)}_\nu )$ on the motion of hidden states and causes. It is these that make the model probabilistic—they play the role of sensory noise at the first level and induce uncertainty about states at higher levels. The precisions of these fluctuations are quantified by $(\Pi ^{(i)}_x, \Pi ^{(i)}_\nu )$ which are defined as the inverse of the respective covariance matrices.

The deterministic part of the model is specified by nonlinear functions of hidden states and causes $(f^{(i)} , g^{(i)})$ that generate dynamics and sensory consequences. Hidden causes link hierarchical levels, whereas hidden states link dynamics over time. Hidden states and causes are abstract quantities that the brain uses to explain or predict sensations—like the motion of an object in the field of view. In hierarchical models of this sort, the output of one level acts as an input to the next. This input can produce complicated convolutions with deep (hierarchical) structure. We will see examples of this later in particular in the context of anticipatory movements.

### Perception and predictive coding

Given the form of the generative model (Eq. 2), one can write down the differential equations (Eq. 1) describing neuronal dynamics in terms of prediction errors on the hidden causes and states. These errors represent the difference between conditional expectations and predicted values, under the generative model (using $A \cdot B := A^T B$ for the scalar product and omitting higher-order terms):

3 $$\begin{aligned} \dot{\tilde{\mu }}^{(i)}_x&= \fancyscript{D} \tilde{\mu }^{(i)}_x + \frac{\partial \tilde{g}^{(i)}}{\partial \tilde{\mu }^{(i)}_x} \cdot \Pi ^{(i)}_\nu \tilde{\varepsilon }^{(i)}_\nu \nonumber \\&+ \frac{\partial \tilde{f}^{(i)}}{\partial \tilde{\mu }^{(i)}_x} \cdot \Pi ^{(i)}_x \tilde{\varepsilon }^{(i)}_x - \fancyscript{D} \Pi ^{(i)}_x \tilde{\varepsilon }^{(i)}_x \nonumber \\ \dot{\tilde{\mu }}^{(i)}_\nu&= \fancyscript{D} \tilde{\mu }^{(i)}_\nu + \frac{\partial \tilde{g}^{(i)}}{\partial \tilde{\mu }^{(i)}_\nu } \cdot \Pi ^{(i)}_\nu \tilde{\varepsilon }^{(i)}_\nu \nonumber \\&+ \frac{\partial \tilde{f}^{(i)}}{\partial \tilde{\mu }^{(i)}_\nu } \cdot \Pi ^{(i)}_x \tilde{\varepsilon }^{(i)}_x - \Pi ^{(i+1)}_\nu \tilde{\varepsilon }^{(i+1)}_\nu \nonumber \\ \tilde{\varepsilon }^{(i)}_x&= \fancyscript{D} \tilde{\mu }^{(i)}_x - \tilde{f}^{(i)}\left( \tilde{\mu }^{(i)}_x, \tilde{\mu }^{(i)}_\nu \right) \nonumber \\ \tilde{\varepsilon }^{(i)}_\nu&= \tilde{\mu }^{(i-1)}_\nu - \tilde{g}^{(i)}\left( \tilde{\mu }^{(i)}_x, \tilde{\mu }^{(i)}_\nu \right) \end{aligned}$$

The quantities $\tilde{\varepsilon }^{(i)}$ correspond to prediction errors (on hidden states $x$ or hidden causes $\nu$). These are weighted by their respective precision vectors $\Pi ^{(i)}$ in the update scheme. Equation 3 can be derived fairly easily by computing the free energy for the hierarchical model in Eq. 2 and inserting its gradients into Eq. 1. This gives a relatively simple update scheme, in which conditional expectations are driven by a mixture of prediction errors, where prediction errors are defined by the equations of the generative model.

It is difficult to overstate the generality and importance of Eq. 3—its solutions grandfather nearly every known statistical estimation scheme, under parametric assumptions about additive noise (Friston 2008). These range from ordinary least squares to advanced variational deconvolution schemes. In this form, one can see clearly the relationship between predictive coding and Kalman–Bucy filtering—changes in conditional expectations comprise a prediction (first term) plus a weighted mixture of prediction errors (remaining terms). The weights play the role of a Kalman gain matrix and are based on the gradients of the model functions and the precision of random fluctuations.

**Fig. 3:**
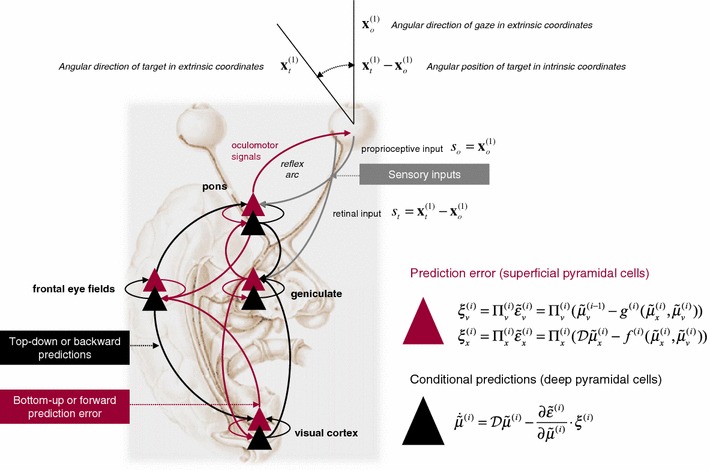
Schematic detailing a neuronal message passing scheme (generalised Bayesian filtering or predictive coding) that optimises conditional expectations about hidden states of the world, given sensory (visual) data and the active (oculomotor) sampling of those data. This *diagram* shows the speculative cells of origin of forward driving connections (in *red*) that convey prediction error from a lower area to a higher area and the backward connections (in *black*) that construct predictions (Mumford 1992). These predictions try to explain away prediction error in lower levels. In this scheme, the sources of forward and backward connections are superficial (*red*) and deep (*black*) pyramidal cells, respectively. The equations on the *right* represent a generalised descent on free energy under the hierarchical model described in the main text—this can be regarded as a generalisation of predictive coding or Kalman filtering: see Friston (2008). State units are in *black* and error units are in *red*. Here, we have placed different levels of some hierarchical model within the visual–oculomotor system. Visual input arrives in an intrinsic (retinal) frame of reference that depends upon the angular position of a stimulus and the direction of gaze. Exteroceptive input is then passed to the lateral geniculate nuclei (LGN) and to higher visual and prefrontal (e.g. motion sensitive, such as the frontal eye field) areas in the form of prediction errors. Crucially, proprioceptive sensations are also predicted, creating prediction errors at the level of the cranial nerve nuclei (pons). The special aspect of these proprioceptive prediction errors is that they can be resolved through classical reflex arcs—in other words, they can elicit action to change the direction of gaze and close the visual–oculomotor loop

In neural network terms, Eq. 3 says that error units receive predictions from the same hierarchical level and the level above. Conversely, conditional expectations (encoded by the activity of state units) are driven by prediction errors from the same level and the level below. These constitute bottom-up and lateral messages that drive conditional expectations towards a better prediction to reduce the prediction error in the level below. This is the essence of recurrent message passing between hierarchical levels to suppress free energy or prediction error: see Friston and Kiebel (2009) for a more detailed discussion. In neurobiological implementations of this scheme, the sources of bottom-up prediction errors, in the cortex, are thought to be superficial pyramidal cells that send forward connections to higher cortical areas. Conversely, predictions are conveyed from deep pyramidal cells by backward connections, to target (polysynaptically) the superficial pyramidal cells encoding prediction error (Friston and Kiebel 2009; Mumford 1992). This defines an elementary circuit that may be the basis of the layered organisation of the cortex (Bastos et al. 2012). Figure 3 provides a schematic of the proposed message passing among hierarchically deployed cortical areas.

### Action

In active inference, conditional expectations elicit behaviour by sending predictions down the hierarchy to be unpacked into proprioceptive predictions at the level of (pontine) cranial nerve nuclei and spinal cord. These engage classical reflex arcs to suppress proprioceptive prediction errors and produce the predicted motor trajectory

4 $$\begin{aligned} \dot{a} = - \partial _a F = -(\partial _a \tilde{\varepsilon }^{(1)}_\nu ) \cdot \Pi ^{(1)}_\nu \tilde{\varepsilon }^{(1)}_\nu \end{aligned}$$

The reduction of action to classical reflexes follows because the only way that action can minimise free energy is to change sensory (proprioceptive) prediction errors by changing sensory signals. This highlights the tight relationship between action and perception; cf., the equilibrium point formulation of motor control (Feldman and Levin 1995). In short, active inference can be regarded as equipping a generalised predictive coding scheme with classical reflex arcs: see Friston et al. (2010b) and Friston et al. (2009) for details. The actual movements produced clearly depend upon (changing) top-down predictions that can have a rich and complex structure. This scheme is consistent with the physiology and anatomy of the oculomotor system (for a review see Ilg 1997; Krauzlis 2004), although our goal here is not to identify the role of each anatomical structure but rather to give a schematic proof-of-concept.

### Summary

In summary, we have derived equations for the dynamics of perception and action using a free energy formulation of adaptive (Bayes optimal) exchanges with the world and a generative model that is both generic and biologically plausible. A technical treatment of the material above will be found in Friston et al. (2010a), which provides the details of the generalised filtering used to produce the simulations in the next section. Before looking at these simulations, we consider how delays can be incorporated into this scheme.

## Active inference with sensorimotor delays

If action and sensations were not subject to delays, one could integrate (solve) eq. 1 directly; however, in the presence of sensory and motor delays ($\tau _s$ and $\tau _a$, respectively), eq. 1 becomes a (stochastic and nonlinear) delay differential equation because $\tilde{s}(t) = \varvec{\tilde{s}}(t - \tau _s)$ and $a(t) = \varvec{a}(t + \tau _a)$. In other words, the agent receives sensations from (sees) the past, while emitting motor signals that will be enacted in the future (we will only consider delays from the sensory and motor sub-systems and neglect delays between neuronal systems in this paper).

To finesse the integration of these delay differential equations, one can exploit their formulation in generalised coordinates: By taking linear mixtures of generalised motion, one can easily map from the present to the future, using the matrix operators:

5 $$\begin{aligned} T(\tau ) = \exp (\tau \fancyscript{D})&= \left[ \begin{array}{c@{\quad }c@{\quad }c@{\quad }c}1 &{} \frac{1}{1!}\tau &{} \frac{1}{2!}\tau ^2 &{} \ldots \\ 0 &{} 1 &{} \frac{1}{1!}\tau &{} 0 \\ 0 &{} 0 &{} 1 &{} \ddots \\ 0 &{} 0 &{} 0 &{} \ddots \end{array}\right] \nonumber \\ \text {with } \fancyscript{D}&= \left[ \begin{array}{c@{\quad }c@{\quad }c@{\quad }c} 0 &{} 1 &{} 0 &{} 0\\ 0 &{} 0 &{} 1 &{} 0 \\ 0 &{} 0 &{} 0 &{} \ddots \\ 0 &{} 0 &{} 0 &{} 0 \end{array}\right] \end{aligned}$$

The first differential operator simply returns the generalised motion $\fancyscript{D} \tilde{x}(t) = \tilde{x}^{\prime }(t)$ while the second delay operator produces generalised states in the future $T(\tau ) \tilde{x}(t) = \tilde{x}(t+\tau )$ (we define delays as positive by convention). Note that shifting forwards and backwards by the same amount of time produces the identity operator $T(\tau ) T(-\tau ) = I$ and that, more generally, $T(\tau _1) T(\tau _2) = T(\tau _1 + \tau _2)$.

These delay operators are simple to implement computationally (and neurobiologically) and allow an agent to finesse the delayed coupling above by replacing (delayed) sensory signals with future input $\tilde{s}(t)=T(\tau _s)\varvec{\tilde{s}}(t-\tau _s) = \varvec{\tilde{s}}(t)$ for subsequent action and perception. Alternatively, one can regard this compensation for sensory delays as attempting to predict the past (see below). Generalised coordinates allow the representation of the trajectory of a given variable at any time (that is its evolution in the near past and present) and thus allow its projection into the future or past. Generalised representations are more extensive than ‘snapshots’ at a particular time and enable the agent to anticipate the future (of delayed sensory trajectories) and represent hidden states in real time—that is, representations that are synchronised with the external events. In terms of motor delays, the agent can replace its internal motor signals with action in the future $\varvec{a}(t) = T(\tau _a) a(t-\tau _a) = a(t)$, such that when action signals reach the periphery, they correspond to the action encoded centrally. These substitutions allow us to express action and perception in Eq. 1 as^1^:

6 $$\begin{aligned} \varvec{\dot{a}}(t) \!&= \! -\partial _a F(T(\tau _a) T(\tau _s) \varvec{\tilde{s}}(t\!-\varvec{\tau }_s\!-\!\varvec{\tau }_a),\quad \! T(\tau _a) \tilde{\mu }(t\!-\!\varvec{\tau }_a))\nonumber \\ \!&= \! -\partial _a F(T(\tau _s\!-\varvec{\tau }_s\!+\!\tau _a-\varvec{\tau }_a) \varvec{\tilde{s}}(t),\quad \! T(\tau _a-\varvec{\tau }_a) \tilde{\mu }(t))\nonumber \\ \dot{\tilde{\mu }}(t)&= \fancyscript{D}\tilde{\mu }(t)-\partial _{\tilde{\mu }} F(T(\tau _s) \varvec{\tilde{s}}(t-\varvec{\tau }_s),\quad \tilde{\mu }(t))\nonumber \\&= \fancyscript{D}\tilde{\mu }(t)-\partial _{\tilde{\mu }} F(T(\tau _s-\varvec{\tau }_s) \varvec{\tilde{s}}(t),\quad \tilde{\mu }(t)) \end{aligned}$$

This equation distinguishes between true delays ($\varvec{\tau }$) and those assumed by the agent ($\tau$). When the two are the same, the delay operators $T(\tau -\varvec{\tau })=I: \tau =\varvec{\tau }$ become identity matrices and Eq. 6 reduces to Eq. 1. When the two differ, Eq. 6 permits the simulation of a system with uncompensated delays. Notice how the dynamics of action in the first differential equation are driven by a gradient descent on the free energy of sensations with composite sensory and motor delays. In other words, action in the real world depends upon sensory states generated $\varvec{\tau }_s+\varvec{\tau }_a$ in the past.

One can now solve eq. 6 to simulate active inference, with or without compensation for sensorimotor delays. We use a standard local linearisation scheme for this integration (Ozaki 1992), where delays enter at the point at which sensory prediction error is computed and when it drives action: from Eqs. 3 and 4:

7 $$\begin{aligned} \tilde{\varepsilon }^{(1)}_\nu&= T(\tau _s) \varvec{\tilde{s}}(t-\varvec{\tau }_s) - \tilde{g}^{(1)}(\tilde{\mu }^{(1)}_x, \tilde{\mu }^{(1)}_\nu ) \nonumber \\&= T(\tau _s-\varvec{\tau }_s) \varvec{\tilde{s}}(t) - \tilde{g}^{(1)}(\tilde{\mu }^{(1)}_x, \tilde{\mu }^{(1)}_\nu ) \nonumber \\ \varvec{\dot{a}}(t)&= -(\partial _a \tilde{\varepsilon }^{(1)}_\nu ) \cdot \Pi ^{(1)}_\nu T(\tau _a) \tilde{\varepsilon }^{(1)}_\nu (t-\varvec{\tau }_a) \nonumber \\&= -(\partial _a \tilde{\varepsilon }^{(1)}_\nu ) \cdot \Pi ^{(1)}_\nu T(\tau _a-\varvec{\tau }_a) \tilde{\varepsilon }^{(1)}_\nu (t) \end{aligned}$$

Equation 7 means that perfect (errorless) prediction requires $T(\tau _s) \varvec{\tilde{s}}(t-\varvec{\tau }_s) = \tilde{g}^{(1)}(\tilde{\mu }^{(1)}_x, \tilde{\mu }^{(1)}_\nu )$. In other words, errorless prediction means that the agent is effectively predicting the future projection of the past. Note again the dependency of action, via prediction errors, on sensory states $\varvec{\tau }_s+\varvec{\tau }_a$ in the past. See Appendix 3 for further details of the integration scheme used in the simulations below.

### Summary

This section has considered how the differential equations describing changes in action and internal (representational) states can be finessed to accommodate sensorimotor delays. This is relatively straightforward—in the context of generalised schemes—using delay operators that take mixtures of generalised motion to project states into the future or past. Sensory delays can be (internally) simulated and corrected by applying delays to sensory input producing sensory prediction error, while motor delays can be simulated and corrected by applying delays to sensory prediction error producing action. Neurobiologically, the application of delay operators just means changing synaptic connection strengths to take different mixtures of generalised sensations and their prediction errors. We will now use these operators to look at the effects of sensorimotor delays with and without compensation.

## Results: pursuit initiation

This section focuses on the consequences of sensory delays, motor delays and their combination—in the context of pursuit initiation—using perhaps the simplest generative model for active inference. Our purpose is to illustrate the difficulties in oculomotor control that are incurred by delays and how these difficulties dissolve when delays are accommodated during active inference. We start with a description of the generative model and demonstrate its behaviour when delays are compensated. We then use this normal behaviour as a reference to look at failures of pursuit initiation induced by delays. In this section, responses to a single sweep of rightward motion are used to illustrate basic responses. In the next section, we consider pursuit of sinusoidal motion (with abrupt onsets) and the implications for generative models that may be used by the brain.

### Generative model of pursuit initiation

The generative model for pursuit initiation used here is very simple and is based upon the prior belief that the centre of gaze is attracted to the target location. The processes generating sensory inputs and the associated generative model can be expressed as follows:

8 $$\begin{aligned}&\varvec{s} = \left[ \begin{array}{c} \varvec{s}_o \\ \varvec{s}_t \end{array}\right] = \left[ \begin{array}{c} \varvec{x}_o \\ \varvec{x}_t - \varvec{x}_o \end{array}\right] + \varvec{\omega }^{(1)}_\nu \nonumber \\&\varvec{\dot{x}} = \left[ \begin{array}{c} \varvec{\dot{x}}_o \\ \varvec{\dot{x}}_t \end{array}\right] = \left[ \begin{array}{c} \frac{1}{t_a} a-\frac{1}{t_o}\varvec{x}_o \\ \frac{1}{t_m}(\varvec{\nu }^{(1)} - \varvec{x}_t) \end{array}\right] + \varvec{\omega }^{(1)}_x \nonumber \\&s = \left[ \begin{array}{c} s_o \\ s_t \end{array}\right] = \left[ \begin{array}{c} x_o \\ x_t - x_o \end{array}\right] + \omega ^{(1)}_\nu \nonumber \\&\dot{x} = \left[ \begin{array}{c} \dot{x}_o \\ \dot{x}_t \end{array}\right] = \left[ \begin{array}{c} \frac{1}{t_s}(x_t-x_o) \\ \frac{1}{t_m}(\nu ^{(1)} - x_t) \end{array}\right] + \omega ^{(1)}_x \nonumber \\&\nu ^{(1)} = \omega ^{(2)}_x \end{aligned}$$

The first pair of equations corresponds to a noisy sensory mapping from hidden states and the equations of motion for states in the real world. These pertain to real-world variables representing the position of the target and of the eye (in boldface). The remaining equations constitute the generative model of how sensations are generated using the form of Eq. 2. These define the free energy in Eq. 1—and specify behaviour under active inference. The variables constitute the first layer of the hierarchical model (see Eq. 2, but for simplicity, we have written $\varvec{x}$ instead of $\varvec{x}^{(1)}$ and ${x}$ instead of ${x}^{(1)}$).

The real-world provides sensory input in two modalities: proprioceptive input from cranial nerve nuclei reports the angular displacement of the eye $\varvec{s}_o \in \mathbb {R}^2$ and corresponds to the centre of gaze. Note that, using the approximation of relatively small displacements, we use Cartesian coordinates to follow previous treatments, e.g. Friston et al. (2010a). However, visual space is better described by bounded polar coordinates, and treatments of large eye movements should account for this. Exteroceptive (retinal) input reports the angular position of a target in a retinal (intrinsic) frame of reference $\varvec{s}_t \in \mathbb {R}^2$. The indices $o$ and $t$ thus refer to states of the oculomotor system or of the target, respectively. Note that $\varvec{s}_t$ is just the difference between the centre of gaze and target location in an extrinsic frame of reference $\varvec{x}_t - \varvec{x}_o$. In this paper, we are modelling the online inference of target position, and we are ignoring the problem of how the causal structure of the environment is learned. We simply assume that this structure has already been learned accurately, and therefore, the dynamics of the real world and the generative model are the same. Clearly, this model of visual processing is an enormous simplification: we are assuming that place coded spatial information can be summarised in terms of displacement vectors. However, more realistic simulations—using a set of retinotopic inputs with classical receptive fields covering visual space—produce virtually the same results. We will use more realistic models in future publications that deal with smooth pursuit and visual occlusion. Here, we use the simpler formulation to focus on delays and the different sorts of generative models that can provide top-down or extra-retinal constraints on visual motion processing.

The hidden states of this model comprise the true, real-world oculomotor displacement ($\varvec{x}_o \in \mathbb {R}^2$) and target location ($\varvec{x}_t \in \mathbb {R}^2$). The units of angular displacement are arbitrary, but parameters are tuned to correspond to a small displacement of 4 degrees of visual angle for one arbitrary unit (that is approximately 4 times the width of a thumb’s nail at arm’s length). The oculomotor state is driven by action with a time constant of $$t_a=64~\hbox {ms}\ $$ and decays (slowly) to zero through damping, with a time constant of $t_o = 512~\hbox {ms}$. The target location is perturbed by hidden causes $\varvec{x}_t \in \mathbb {R}^2$ that describe the location to which the target is drawn, with a time constant of $t_m=16~\hbox {ms}$. In this paper, the random fluctuations on sensory input and on the motion of hidden states are very small, with a log precision of 16. In other words, the random fluctuations have a variance of $\exp (-16)$. This completes our description of the process generating sensory information, in which hidden causes force the motion of a target location and action forces oculomotor states. Target location and oculomotor states are combined to produce sensory information about the target in an intrinsic frame of reference.

The generative model has exactly the same form as the generative process but with one important exception: there is no action and the motion of the hidden oculomotor states is driven by the displacement between the target location and the central gaze (with a time constant of $t_s=32~\hbox {ms}$). In other words, the agent believes that its gaze will be attracted to the location of the target, which, itself, is being driven by some unknown exogenous force or hidden cause. The log precisions on the random fluctuations in the generative model were four, unless stated otherwise. This means that uncertainty about sensory input, (motion of) hidden states and causes was roughly equivalent.

Having specified the generative process and model, we can now solve the active inference scheme in Eq. 1 and examine its behaviour. Sensorimotor delays are implemented in the message passing from the generative process to the generative model. This generative model produces pursuit initiation because it embodies prior beliefs that the centre of gaze will follow the target location. This pursuit initiation rests on conditional expectations about the target location in extrinsic coordinates and the state of the oculomotor plant, where the location is driven by hidden causes that also have to be inferred.

The generative model described in this section provides the equations required to simulate active inference using the formalism of the previous section. In short, we now consider the generative model that defines the variational free energy and (Bayes) optimal active inference.

### Simulations

**Fig. 4:**
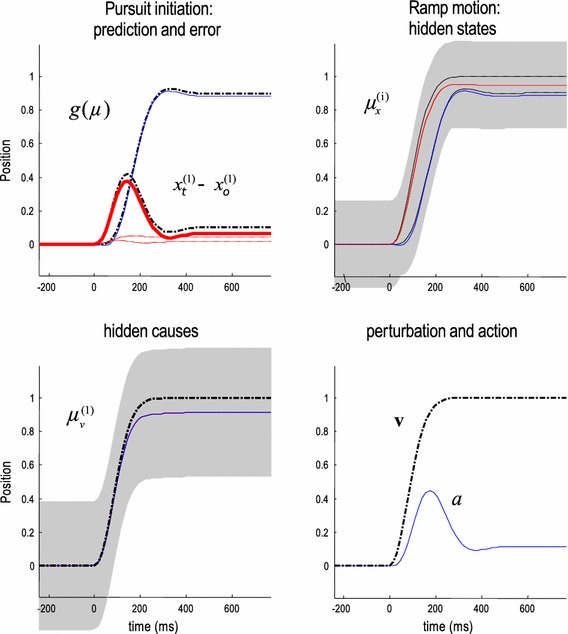
This figure reports the conditional estimates of hidden states and causes during the simulation of pursuit initiation, using a single rightward (positive) sweep of a visual target, while compensating for sensory motor delays. We will use the format of this figure in subsequent figures: the *upper left panel* shows the predicted sensory input (*coloured lines*) and sensory prediction errors (*dotted red lines*) along with the true values (*broken black lines*). Here, we see horizontal excursions of oculomotor angle (*upper lines*) and the angular position of the target in an intrinsic frame of reference (*lower lines*). This is effectively the distance of the target from the centre of gaze and reports the spatial lag of the target that is being followed (*solid red line*). One can see clearly the initial displacement of the target that is suppressed after a few hundred milliseconds. The sensory predictions are based upon the conditional expectations of hidden oculomotor (*blue line*) and target (*red line*) angular displacements shown on the upper right. The *grey regions* correspond to 90 % Bayesian confidence intervals and the *broken lines* show the true values of these hidden states. One can see the motion that elicits following responses and the oculomotor excursion that follows with a short delay of about 64 ms. The hidden cause of these displacements is shown with its conditional expectation on the *lower left*. The true cause and action are shown *on the lower right*. The action (*blue line*) is responsible for oculomotor displacements and is driven by the proprioceptive prediction errors

All simulations were performed with a time bin of 16ms, and we report results in milliseconds. All results were replicated with different time bins (16ms, 8ms, 4ms, 2ms and 1ms) with minimal changes to the results. Figure 4 reports the conditional estimates of hidden states and causes during the simulation of pursuit initiation, using a simple rightward sweep of a visual target and compensating for sensorimotor delays: $\tau _s = \varvec{\tau }_s$ and $\tau _a = \varvec{\tau }_a$. This compensation is effectively the same as simulating responses in the absence of delays—because the delay operators reduce to the identity matrix. Target motion was induced using a hidden cause that was a ramp function of post-stimulus time. Note that ramp stimuli are often used in psychophysics, and this generative model—using velocity in place of position—produces the same results in velocity space. Indeed, most models, such as Robinson et al. (1986) or Krauzlis and Lisberger (1989), focus on modelling velocity responses. We choose to model the tracking of position for two reasons: First, it is easy to generalise position results to velocity using generalised coordinates of motion. Second, positional errors can induce slow eye movements (Kowler and Steinman 1979; Wyatt and Pola 1981) and we hoped to accommodate this in the model. If we assume that the units of angular displacement are 4 degrees of visual angle, then the resulting peak motion corresponds to about 20 degrees per second.

The upper left panel shows the predicted sensory input (coloured lines) and sensory prediction errors (dotted red lines) along with the true values (broken black lines). Here, we see horizontal excursions of oculomotor angle (upper lines) and the angular position of the target in an intrinsic frame of reference (lower lines). This is effectively the distance of the target from the centre of gaze and reports the spatial lag of the target that is being followed (solid red line). One can see clearly an initial retinal displacement of the target that is suppressed after approximately $20~\hbox {ms}$. This effect confirms that the visual representation of target position is predictive and that the presentation of a smooth predictable versus an unpredictable target would induce a lag between their relative positional estimates, as is evidenced in the *flash-lag effect* (Nijhawan 1994).

**Fig. 5:**
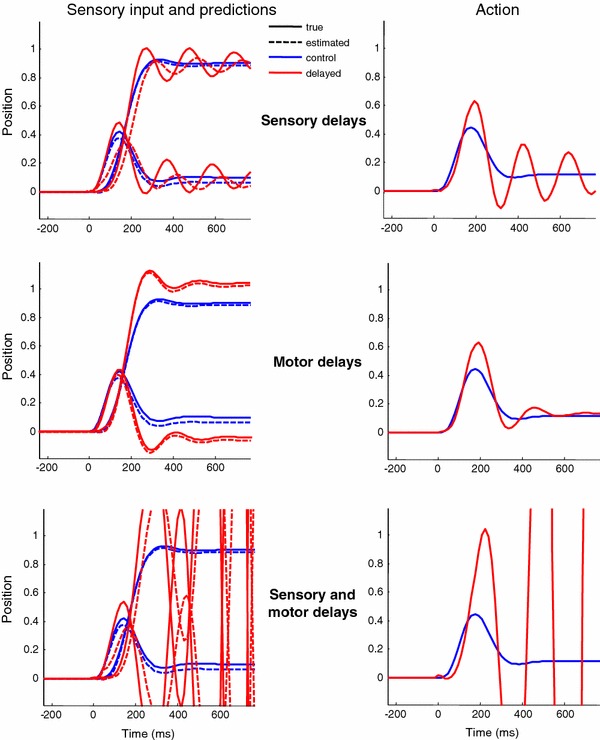
This figure illustrates the effects of sensorimotor delays on pursuit initiation (*red lines*) in relation to compensated (optimal) active inference—as shown in the previous figure (*blue lines*). The *left panels* show the true (*solid lines*) and estimated sensory input (*dotted lines*), while action is shown in the *right panels*. Under pure sensory delays (*top row*), one can see clearly the delay in sensory predictions, in relation to the true inputs. The thicker (*solid and dotted*) *red lines* correspond, respectively, to (true and predicted) proprioceptive input, reflecting oculomotor displacement. The *middle row* shows the equivalent results with pure motor delays, and the *lower row* presents the results with combined sensorimotor delays. Of note here is the failure of optimal control with oscillatory fluctuations in oculomotor trajectories, which become unstable under combined sensorimotor delays

The sensory predictions are based upon the conditional expectations of hidden oculomotor (blue line) and target (red line) angular displacements shown on the upper right. The grey regions correspond to 90 % Bayesian confidence intervals, and the broken lines show the true values. One can see clearly the motion that elicits pursuit initiation responses, where the oculomotor excursion follows with a short delay of about $64~\hbox {ms}$. The hidden cause of these displacements is shown with its conditional expectation on the lower left. The true cause and action are shown on the lower right. The action (blue line) is responsible for oculomotor displacements and is driven by proprioceptive prediction errors. Action does not return to zero because the sweep is maintained at an eccentric position during this simulation. This eye position slightly undershoots the target position: it is held at around 95 % of the target eccentricity in the upper right panel. Note that this corresponds roughly to the steady-state gain observed in behavioural data, which was modelled explicitly by Robinson et al. (1986). For our purposes, these simulations can be regarded as Bayes optimal solutions to the pursuit initiation problem, in which sensorimotor delays have been accommodated (discounted) via absorption into the generative model. We can now examine the performance in the absence of compensation and see how sensory and motor delays interact to confound pursuit initiation:

The above simulations were repeated with uncompensated sensory delays ($\tau _s =0~\hbox {ms}$ and $\varvec{\tau }_s=32~\hbox {ms}$), uncompensated motor delays ($\tau _a =0~\hbox {ms}$ and $\varvec{\tau }_a=32~\hbox {ms}$) and combined sensorimotor delays of $64~\hbox {ms}$ ($\tau _a =\tau _s =0~\hbox {ms}$ and $\varvec{\tau }_a=\varvec{\tau }_s=32~\hbox {ms}$). To quantify behaviour, we focus on the sensory input and underlying action. The position of the target in intrinsic coordinates corresponds to spatial lag and usefully quantifies pursuit initiation performance. Figure 5 shows the results of these three simulations (red lines) in relation to the compensated (optimal) active inference shown in the previous figure (blue lines). True sensory input corresponds to solid lines and its conditional predictions to dotted lines. The left panels show the true and predicted sensory input, while action is shown in the right panels. Under pure sensory delays (top row), one can see the delay in sensory predictions, in relation to the true inputs. The thicker (solid and dotted) red lines correspond, respectively, to (true and predicted) proprioceptive input, reflecting oculomotor displacement. Crucially, in contrast to optimal control, there are oscillatory fluctuations in oculomotor displacement and the retinotopic location of the target that persists even after the target is stationary. These fluctuations are similar to the oscillations elicited by adding an artificial feed-back delay  (Goldreich et al. 1992). Here, the fluctuations are caused by damped oscillations in action due to, and only to, sensory proprioceptive and exteroceptive delays. These become unstable (increasing in their amplitude) when the predicted value oscillates in counter phase with the real value. Similar oscillations are observed with pure motor delays (middle row). However, here there is no temporal lag between the true and predicted sensations (solid vs. dashed lines). Furthermore, there is no apparent delay in action–action appears to be emitted for longer, reaching higher amplitudes. In fact, action is delayed but the delay is obscured by the increase in the amplitude of action—that is induced by greater proprioceptive prediction errors. If we now combine both sensory and motor delays, we see a catastrophic failure of oculomotor tracking (lower row). With combined sensorimotor delays the pursuit initiation becomes unstable, with exponentially increasing oscillations as action over-compensates for delay-dependent errors.

In effect, the active inference scheme has undergone a phase transition from a stable to an unstable fixed point. We have illustrated this bifurcation by increasing sensorimotor delays under a fixed motor precision or gain in Eq. 7. The results in Fig. 5 used a motor gain with a log precision of 2.5. We chose this value because it produced stable responses with sensory or motor delays alone and unstable dynamics with combined delays. These results illustrate the profound and deleterious effects of sensorimotor delays on simple pursuit initiation, using biologically plausible values—namely sensorimotor delays of 64 ms and a target velocity of about 16 degrees per second. This also illustrates the necessity of compensation for these delays so that the system can achieve a more robust and stable response. One would anticipate, in the face of such failures, real subjects would engage interceptive saccades to catch the target, of the sort seen in schizophrenic patients (Levy et al. 1993). In the remainder of this paper, we will concentrate on the nature of pursuit initiation and smooth pursuit with compensated sensorimotor delays, using a reasonably high motor gain with a log precision of four.

### Pursuit initiation and visual contrast

**Fig. 6:**
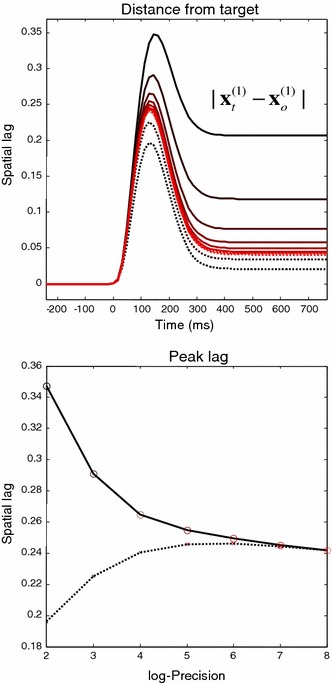
This figure reports the spatial lag (the displacement of the target from the centre of gaze) as a function of contrast (log precision of exteroceptive sensory input). The *upper panel* shows the true (*solid lines*) and predicted (*dotted lines*) spatial lag as a function of peristimulus time for different log precisions, ranging from two (*black lines*) to eight (*red lines*). The peak lags are plotted in the lower panel as a function of visual contrast or log precision. These results show how the perceived lag increases with contrast, while the true lag decreases in accord with empirical observations

Before turning to more realistic generative models of smooth pursuit, we consider the empirical phenomena in which following responses to the onset of target movement are suppressed by reducing the visual contrast of the target (Thompson 1982). In simulations of this sort, visual contrast is modelled in terms of the precision of sensory information in accord with Weber’s law—see Feldman and Friston (2010) for details. Contrast-dependent effects are easy to demonstrate in the context of active inference. Figure 6 shows the spatial lag—the displacement in intrinsic coordinates of the target from the centre of gaze depicted by the solid red line in Fig. 4—as a function of contrast or log precision of exteroceptive sensory input. The upper panel shows the true (solid lines) and predicted (dotted lines) spatial lag as a function of peristimulus time for different log precisions, ranging from two (low) to eight (high). The peak lags are plotted in the lower panel as a function of visual contrast or log precision. Since estimation error decreases as visual contrast increases, both curves converge, leading to a decrease to zero of the prediction error. These results show, in accord with empirical observations, how the spatial lag (position error) increases with contrast (Arnold et al. 2009), while the true lag decreases (Spering et al. 2005). A similar difference between perception and action was recently reported (Simoncini et al. 2012). The explanation for this contrast–dependent behaviour is straightforward—because pursuit initiation is based upon proprioceptive prediction errors, it depends upon precise sensory information. Reducing the precision of visual input—through reducing contrast—increases uncertainty about visual information (sensory estimation error) and places more weight on prior beliefs and proprioceptive sensations. This reduces the perceived motion of the target and reduces the amplitude of prediction errors driving action.

### Summary

In this section, we have seen that sensorimotor delays can have profound and deleterious effects on optimal oculomotor control. Here, optimal control means Bayes optimal active inference, in which pursuit initiation emerges spontaneously from prior beliefs about how a target attracts the centre of gaze. These simulations demonstrate that it is relatively easy to compensate for sensorimotor delays by exploiting representations in generalised coordinates of motion. Furthermore, the resulting scheme has some construct validity in relation to experimental manipulations of the precision or contrast of visual information. However, there are certain aspects of oculomotor tracking that suggest the pursuit initiation model above is incomplete: when presented with periodic target motion, the latency of motor gain (defined operationally in terms of the target and oculomotor velocities) characteristically reduces after the first cycle of target motion (Barnes et al. 2000). This phenomenon cannot be reproduced by the pursuit initiation model above.

**Fig. 7:**
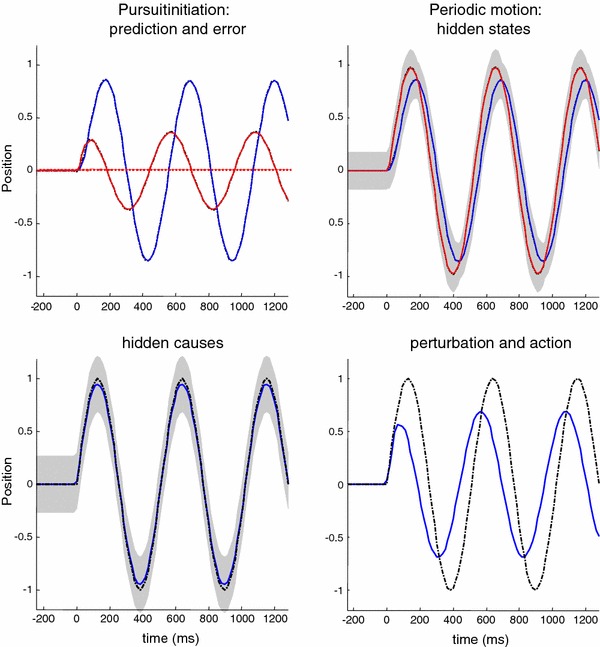
This figure uses the same format as Fig. 4—the only difference here is that the target motion is sinusoidal. The key thing to take from this simulation is that the peak spatial lag at the onset of the second cycle of target motion is greater than the peak lag at the onset of the first. This is contrary to empirical predictions

Figure 7 shows the responses of the pursuit initiation model to sinusoidal motion using the same format as Fig. 4. Here, the hidden cause driving the target was a sine wave with a period of $512~\hbox {ms}$ that started after $256~\hbox {ms}$. If we focus on the spatial lag (solid red line in the upper left panel), one can see that the lag is actually greater after one period of motion than at the onset of motion. This contrasts with empirical observations, which suggest that the spatial lag should be smaller after the first cycle (Barnes et al. 2000). In the next section, we consider a more realistic generative model that resolves this discrepancy and takes us from simple pursuit initiation to smooth pursuit.

## Results: smooth pursuit

In this section, we consider a slightly more realistic generative model that replaces the prior beliefs about the target attracting the centre of gaze with the belief that both the target and centre of gaze are attracted by the same (fictive) location in visual space. This allows pursuit initiation to anticipate the trajectory of the target and pursue the target more accurately—providing the trajectories are sufficiently smooth. The idea behind this generative model is to account for the improvements in tracking performance that are not possible at the onset of motion and that are due to inference on smooth target trajectories.

### Smooth pursuit model

The smooth pursuit model considered in this paper rests on a second-order generalisation of the pursuit initiation model of previous section. Previously, we have considered the motion of the oculomotor plant to be driven directly by action. This form of action can be considered as an (adiabatic) solution to a proper second-order formulation, in which action exerts a force and thereby changes the angular acceleration of oculomotor displacement. This second-order formulation can be expressed in terms of the following generative process and model

9 $$\begin{aligned} \varvec{s}&= \left[ \begin{array}{c} \varvec{s}_o \\ \varvec{s}_t \end{array}\right] = \left[ \begin{array}{c} \varvec{x}_o \\ \varvec{x}_t - \varvec{x}_o \end{array}\right] + \varvec{\omega }^{(1)}_\nu \nonumber \\ \varvec{\dot{x}}&= \left[ \begin{array}{c} \varvec{\dot{x}}_o \\ \varvec{\dot{x}}^{\prime }_o \\ \varvec{\dot{x}}_t \end{array}\right] = \left[ \begin{array}{c} \varvec{x}^{\prime }_t \\ \frac{1}{t_a} a-\frac{1}{t_o}\varvec{x}^{\prime }_o \\ \frac{1}{t_m}(\varvec{\nu }^{(1)} - \varvec{x}_t) \end{array}\right] + \varvec{\omega }^{(1)}_x \nonumber \\ s&= \left[ \begin{array}{c} s_o \\ s_t \end{array}\right] = \left[ \begin{array}{c} x_o \\ x_t - x_o \end{array}\right] + \omega ^{(1)}_\nu \nonumber \\ \dot{x}&= \left[ \begin{array}{c} \dot{x}_o \\ \dot{x}^{\prime }_o \\ \dot{x}_t \end{array}\right] = \left[ \begin{array}{c} {x}^{\prime }_t \\ \frac{1}{t_v}(\nu ^{(1)}-x_o) - \frac{t_s}{t_v}{x}^{\prime }_o \\ \frac{1}{t_m}(\nu ^{(1)} - x_t)\end{array}\right] + \omega ^{(1)}_x \nonumber \\ \nu ^{(1)}&= \omega ^{(2)}_\nu \end{aligned}$$

Here, the only thing that has changed is that we have introduced new hidden states corresponding to oculomotor velocity $\varvec{x}^{\prime }_o \in \mathbb {R}^2$. Action now changes the motion of the velocity (i.e. acceleration), as opposed to the velocity directly. This difference is reflected in the generative model but with one crucial addition—the hidden oculomotor state is not driven by the displacement between the *target* and the centre of gaze but by the displacement between the *hidden cause* and the centre of gaze. In other words, the hidden oculomotor states are attracted by the hidden cause of target motion—not the target motion *per se*. The idea here is that inference about the trajectory of the hidden cause should enable an anticipatory optimisation of pursuit initiation, provided these trajectories are smooth—hence a smooth pursuit model. Note that the equation of motion in the oculomotor model $\dot{x}_o = \frac{1}{t_s}(x_t-x_o)$ (see Eq. 8) is the (adiabatic) solution to the equation used to model smooth pursuit: $\frac{1}{t_v}(\nu ^{(1)}-x_o) - \frac{t_s}{t_v}{x}^{\prime }_o =0$ when $\nu ^{(1)} = x_t$ (see Eq. 9). As a result (and as confirmed by simulations), this model behaved similarly for the sweep stimulus used in Figs. 4, 5 and 6.

### Simulations

**Fig. 8:**
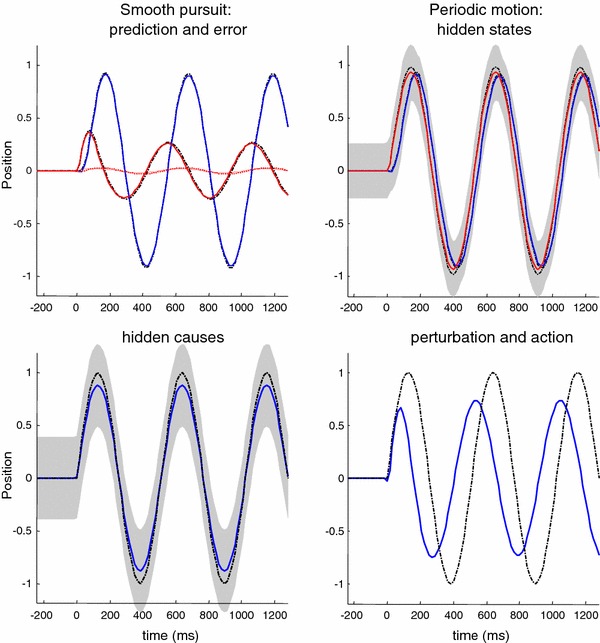
This figure uses the same format as the previous figure—the only difference here is that we have replaced the pursuit initiation model with a smooth pursuit model. In the smooth pursuit model, the centre of gaze is attracted by a hidden cause of target motion, as opposed to the target *per se*. Note that, in comparison with the previous figure, the peak lag at the onset of the second cycle of target motion is now smaller than at the onset to the first

We repeated the simulation reported in Fig. 7 using the smooth pursuit generative model. The results of this simulation are shown in Fig. 8 using the same format as Fig. 7. The key difference—in terms of performance—is that the peak spatial lag after one cycle of motion is now less than the peak lag at the onset of motion. The response to the sinusoid trajectory contrasts with simple pursuit initiation and is more consistent with empirical observations. The true and expected hidden states show that the oculomotor trajectory now follows the target trajectory more accurately, particularly at the peaks of rightward and leftward displacement. Interestingly, the amplitude of action has not changed very much (compare Figs. 7 and 8, upper right panels). However, action is initiated with a slightly shorter latency, which is sufficient to account for the improved pursuit when informed by the prior beliefs about the smooth trajectory of the target.

### Summary

**Fig. 9:**
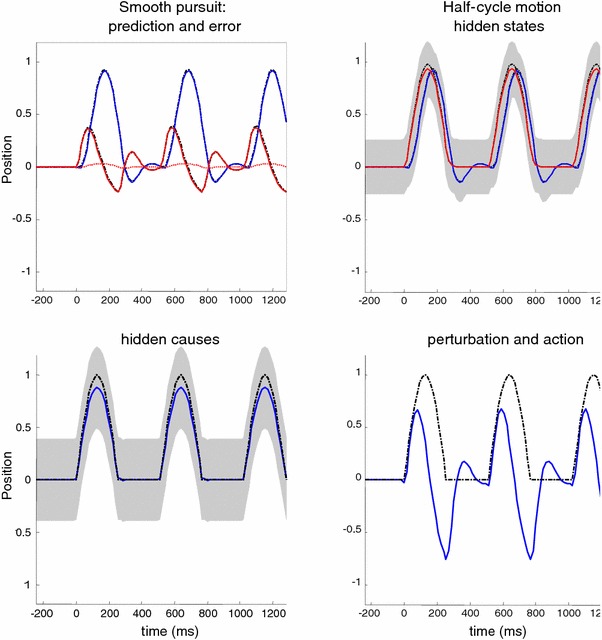
This figure uses the same format as the previous figure—the only difference is that the target motion has been rectified so that it is (approximately) hemi-sinusoidal. The thing to note here is that the improved accuracy of the pursuit previously apparent at the onset of the second cycle of motion has now disappeared—because active inference does not have access to the immediately preceding trajectory. This failure of an anticipatory improvement in tracking is contrary to empirical predictions

In summary, by simply replacing the target with the hidden cause of target motion—as the attractor of oculomotor trajectories—we can account for empirical observations of improved pursuit during periodic target motion. In the context of active inference, this smooth trajectory can only be recognised—and used to inform action—after the onset of periodic motion. However, this smooth pursuit model still fails to account for anticipatory effects that are not directly available in sensory trajectories. Empirical observations suggest that any systematic or regular structure in target motion can facilitate the accuracy of smooth pursuit, even if this information is not represented explicitly in target motion. A nice example of this rests on the use of rectified periodic motion, in which only rightward target excursions are presented. Experimentally, subjects can anticipate the periodic but abrupt onset of motion, provided they recognise the underlying periodic behaviour of the target. We can emulate this hemi-periodic motion by thresholding the hidden cause to suppress leftward deflections. Figure 9 shows the results of simulating smooth pursuit using the same format as Fig. 8. The only difference here is that we replaced the sinusoidal hidden cause $\varvec{\nu }(t) = \sin (2\pi f \cdot t)$ with $\varvec{\nu }(t)= \exp (4(\sin (2\pi f \cdot t)-1))$. This essentially suppresses motion before rightward motion. This suppression completely removes the benefit of smooth pursuit after a cycle of motion—compare Figs. 8 and 9. Here, the peak spatial lag at the onset of the second cycle of motion is exactly the same as the lag at the onset of motion; in other words, there is no apparent benefit of modelling the hidden causes of motion in terms of pursuit accuracy. This failure to model the anticipatory eye movements seen experimentally leads us to consider a full hierarchical model for anticipatory pursuit.

## Results: anticipatory pursuit

This section presents a full hierarchical model of anticipatory smooth pursuit eye movements that tries to account for anticipatory oculomotor responses that are driven by extra-retinal beliefs about the periodic behaviour of targets. This entails adding a hierarchical level to the model that enables the agent to recognise and remember the latent structure in target trajectories and suitably optimise its pursuit movements—which are illustrated here in terms of an improvement in the accuracy of target following after the onset of rectified target motion.

### Anticipatory pursuit

The generative process used in these simulations is exactly the same as in the above (smooth pursuit) scheme (see Eq. 9); however, the generative model of this process is equipped with an extra level in place of the model for the hidden cause of target motion in the generative model:

10 $$\begin{aligned}&s = \left[ \begin{array}{c} s_o \\ s_t \end{array}\right] = \left[ \begin{array}{c} x_o \\ x_t - x_o \end{array}\right] + \omega ^{(1)}_\nu \nonumber \\&\dot{x} = \left[ \begin{array}{c} \dot{x}_o \\ \dot{x}^{\prime }_o \\ \dot{x}_t \end{array}\right] = \left[ \begin{array}{c} {x}^{\prime }_t \\ \frac{1}{t_v}(\nu ^{(1)}-x_o) - \frac{t_s}{t_v}{x}^{\prime }_o \\ \frac{1}{t_m}(\nu ^{(1)} - x_t) \end{array}\right] + \omega ^{(1)}_x \nonumber \\&\nu ^{(1)} = \left[ \begin{array}{c} \sigma (x^{(2)}_1 ) \\ 0 \end{array}\right] + \omega ^{(2)}_\nu \nonumber \\&\dot{x}^{(2)} = \left[ \begin{array}{c} \dot{x}^{(2)}_1 \\ \dot{x}^{(2)}_2 \end{array}\right] = \nu ^{(2)} \left[ \begin{array}{c} x^{(2)}_2 \\ -x^{(2)}_1 \end{array}\right] + \omega ^{(2)}_x \nonumber \\&\nu ^{(2)} = \eta + \omega ^{(3)}_\nu \end{aligned}$$

The first level of the generative model is exactly the same as above. However, the hidden causes are now informed by the dynamics of hidden states at the second level. These hidden states model underlying periodic dynamics using a simple periodic attractor that produces sinusoidal fluctuations of any amplitude or phase and a frequency that is determined by a second-level hidden cause with a prior expectation of a frequency of $\eta$ (in Hz). It is somewhat similar to a control system model that attempted to achieve zero-latency target tracking by fitting the trajectory to a (known) periodic signal (Bahill and McDonald 1983). Our formulation ensures a Bayes optimal estimate of periodic motion in terms posterior beliefs about its frequency. In these simulations, we used a fixed Gaussian prior centred on the correct frequency with a period of $512~\hbox {ms}$. This prior reproduces a typical experimental setting in which the oscillatory nature of the trajectory is known, but its amplitude and phase (onset) are unknown. Indeed, it has been shown that anticipatory responses are cofounded when randomising the inter-cycle interval (Becker and Fuchs 1985). In principle, we could have considered many other forms of generative model, such as models with prior beliefs about continuous acceleration (Bennett et al. 2010).

As above, all the random fluctuations were assumed to have a log precision of four. Crucially, the mapping between the second-level (latent) hidden states and the motion of first-level hidden states encoding trajectories in visual (extrinsic) space is nonlinear. This means that latent periodic motion can be distorted in any arbitrary way. Here, we use a soft thresholding function $\sigma (x) = \exp (4(x-1))$ to suppress negative (rightward) excursions of the target to model hemi-sinusoidal motion. This is the same function we used to generate the motion in Fig. 9. Note that if the precision of the noise at the second level falls to zero and there is no (precise) information at this level, the generative model assumes that the random fluctuations have an infinite variance. As a consequence, the prediction at the level below in the hierarchical model simplifies to $\nu ^{(1)} = \omega ^{(2)}_\nu$, and we recover eq. 9 describing the smooth pursuit model. As a consequence, this parameter tunes the relative strength of anticipatory modulation.

**Fig. 10:**
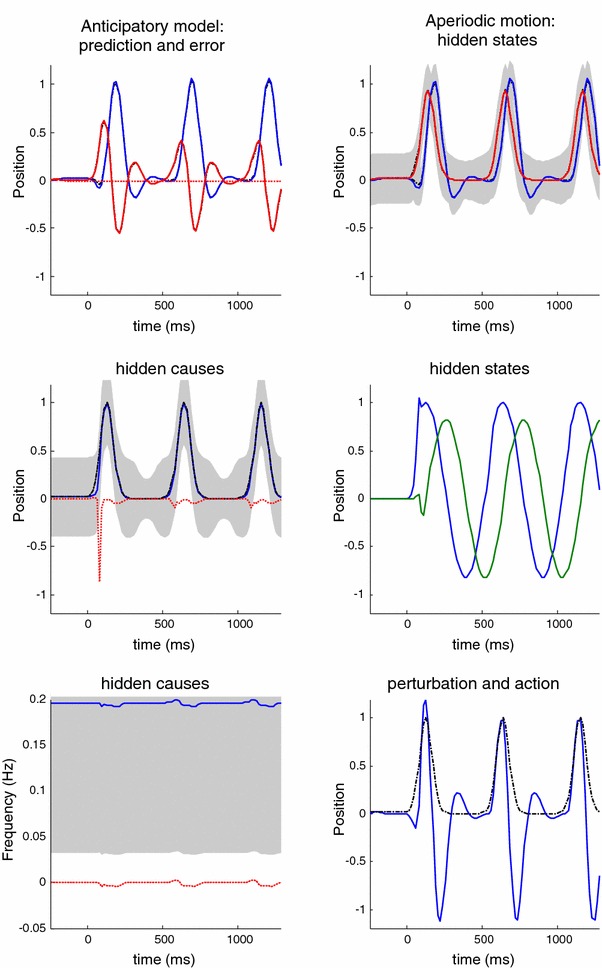
This figure uses the same format as the previous figure—the only difference is that the generative model has been equipped with a second hierarchical level that contains hidden states, modelling latent periodic behaviour of the (hidden) causes of target motion. With this addition, the improvement in pursuit accuracy apparent at the onset of the second cycle of motion is reinstated. This is because the model has an internal representation of latent causes of target motion that can be called upon even when these causes are not expressed explicitly in the target trajectory

Figure 10 shows the results of simulating active inference under this anticipatory model, using the same format as Fig. 9. However, there is now an extra level of hidden states encoding latent periodic motion. It can be seen that expectations about hidden states attain nonzero amplitudes shortly after motion onset and are periodic thereafter. These provide predictions about the onset of rightward motion after the first (latent) cycle, enabling a more accurate oculomotor response. This is evidenced by the reduction in the spatial lag at the onset of the second cycle of motion, relative to the first (solid red lines on the upper left). This improvement in accuracy should be compared to the previous figure and reflects Bayes optimal anticipatory responses of the sort observed empirically (Barnes et al. 2000). Further evidence of anticipatory inference can be seen by examining the conditional expectations about hidden causes at the second level. Note the substantial reduction in prediction error on the hidden cause (dotted red lines), when comparing the onset of the second cycle to the onset of the first. This reflects the fact that the conditional expectations about the hidden cause show a much reduced latency at the onset of the second cycle due to top-down conditional predictions provided by the second-level hidden states. This recurrent and hierarchically informed inference provides the basis for anticipatory oculomotor control and may be a useful metaphor for the hierarchical anatomy of the visual–oculomotor system.

### Summary

In conclusion, to account for anticipatory pursuit movements that are not immediately available in target motion, one needs to equip generative models with a hierarchal structure that can accommodate latent dynamics—that may or may not be expressed at the sensory level. It is important to note that this model is a gross simplification of the complicated hierarchies that may exist in the brain. For instance, while some anticipation may be induced in smooth pursuit eye movements, some aspects, such as the aperture problem, may not be anticipated (Montagnini et al. 2006). In this model, the second-level hidden causes are simply driven by prediction errors and assume a constant frequency. As a consequence, prior beliefs about frequency are modelled as stationary. In the real brain, one might imagine that models of increasing hierarchical depth might allow for nonstationary frequencies and other dynamics—that would better fit behavioural data. We have chosen to illustrate the basic ideas using a minimalistic example of anticipation in eye movements. Hierarchical extensions of this sort emphasise the distinction between visual motion processing and attending oculomotor control based purely upon retinal and proprioceptive input—they emphasise extra-retinal processing that is informed by prior experience and beliefs about the latent causes of visual input. We will exploit this anticipatory smooth pursuit model in future work, where visual occluders are used to disclose beliefs about latent motion.

## Discussion

In this paper, we have considered optimal motor control in the context of pursuit initiation and anticipatory smooth pursuit. In particular, we have taken a Bayesian perspective on optimality and have simulated various aspects of eye movement control using predictive coding and active inference. This provides a solution to the problem of sensorimotor delays that reproduces the results of earlier solutions—but using a neuronally plausible (predictive coding) scheme that has been applied to a whole range of perceptual, psychophysical, decision theoretic and motor control problems beyond oculomotor control. Active inference depends upon a generative model of stimulus trajectories and their active sampling through movement. This requires a careful consideration of the generative models that might be embodied by the visual–oculomotor system—and the sorts of behaviours one would expect to see under these models. The treatment in this paper distinguishes between three levels of predictive coding with respect to oculomotor control: the first is at the lowest level of sensorimotor message passing between the sensorium and internal states representing the causes of sensory signals. Here, we examined the potentially catastrophic effects of sensorimotor delays and how they can easily render oculomotor tracking inherently unstable. This problem can be finessed—in a relatively straightforward way—by exploiting representations in generalised coordinates of motion. These can be used to offset both sensory and motor delays, using simple and neurobiologically plausible mixtures of generalised motion. We then motivated a model of smooth pursuit eye movements by noting that a simple model of target following cannot account for the improvement in visual tracking after the onset of smooth and continuous target trajectories. In this paper, smooth pursuit was modelled in terms of hidden causes that attracted both the target and centre of gaze simultaneously—enabling the trajectory of the target to inform estimates of the hidden cause that, in turn, provide predictions about oculomotor consequences. While this extension accounted for experimentally observed tracking improvements—under continuous trajectories—it does not account for anticipatory movements that have to accumulate information over time. This anticipatory behaviour could only be explained with a deeper hierarchical model that has an explicit representation of latent (periodic) structure causing target motion. When the generative model was equipped with a deeper structure, it was then able to produce anticipatory movements of the sort seen experimentally. Clearly, the simulations in this paper are just heuristic and do not represent a proper simulation of neurobiological processing. However, they can be taken as proof of principle that the basic computational architecture—in terms of generalised representations and hierarchical models—can explain some important and empirical facts about eye movements. In what follows, we consider the models in this paper in relation to other models and how modelling of this sort may have important implications for understanding the visual–oculomotor system.

### Comparison with other models

The model that we have presented here speaks to and complements several existing models of the oculomotor system. First, it shares some properties with computer vision algorithms used for image stabilisation. Such models often use motion detection coupled with salient feature detection for the registration of successive frames (Lucas and Kanade 1981). A major difference is that these models are often applied to very specific problems or configurations for which they give an efficient, yet *ad hoc* solution. A more generic approach is to use—as our model does—a probabilistic method, for instance particle filtering (Isard and Blake 1998). Our model provides a constructive extension—as we integrate the dynamics of both sensation and action. In principle, this could improve the online response of feature tracking algorithms.

Second, using our modelling approach, we reproduce similar behaviours shown by other neuromimetic models of the oculomotor system. For example, the pursuit of a dot with known uncertainty can be modelled as the response of a Kalman filter (Kalman 1960). Both generalised Bayesian (active inference) and Kalman filtering predict the current state of the system using prior knowledge (about previous target locations) and refine these predictions using sensory data (prediction errors). This analogy with block diagrams from control theory was first highlighted by Robinson et al. (1986) and Krauzlis and Lisberger (1989)—and has since been used widely (Grossberg et al. 1997). For a recent treatment involving the neuromorphic modelling of cortical areas, see Shibata et al. (2005). However, it should be noted that the link with Kalman filtering is rarely explicit (but see de Xivry et al. 2013); most models have been derived heuristically, rather than as optimal solutions under a generative model. One class of such neuromimetic models uses neural networks that mimic the behaviour of the Kalman filter (Haykin 2001). This model was used to fit and predict the response of smooth pursuit eye movements under different experimental parameters (Montagnini et al. 2007) or while interrupting information flow (Bogadhi et al. 2011a). Developing this methodology—and by analogy with modular control theory architectures—these building blocks can be assembled to accommodate increasingly complex behavioural tasks. This can take the form of a multi-layered model for transparency processing (Raudies et al. 2011) or of an interconnected graph connecting the form and motion pathways (Beck et al. 2008). Such models have been used to understand adaptation to blanking periods and to tune the balance between sensory and proprioceptive inputs (Madelain and Krauzlis 2003). Our model is different in a key aspect: The Kalman filter is indeed the (Bayes) optimal solution under a linear generative model, but a cascade of such solutions is not the optimal solution to (nonlinear) hierarchical models (Balaji and Friston 2011). The active inference approach considers the (embodied) system as a whole and furnishes an optimal solution in the form of generalised Bayesian filtering. In particular, given the delays at the sensory and motor levels, it provides an optimal solution that accommodates (or compensates for) these delays. As shown in the results, the ensuing behaviour reproduces experimental results from pursuit initiation (Masson et al. 2010) to anticipatory responses (Avila et al. 2006; Barnes et al. 2000). The approach thus provides in inclusive framework, compared with heuristics used in neuromimetic models that focus on specific aspects of oculomotor control (see below).

The model presented here shares many features with other probabilistic models. First, representations are encoded as probability density is. This allows processing and control to be defined in terms of probabilistic inference; for instance, by specifying a prior belief that favours slow speeds (Weiss et al. 2002). This approach has been successful in explaining a wide variety of physiological and psychophysical results. For example, it allows one to model spatial (Perrinet and Masson 2007) or temporal (Montagnini et al. 2007) integration of information, using conditional independence assumptions. Furthermore, recent developments have addressed the estimation of the shape and parameters of priors for slow speeds (Stocker and Simoncelli 2006) and for the integration of ambiguous versus non-ambiguous information (Bogadhi et al. 2011b). The active inference scheme used here relies on generative models that entail exactly the same sorts of priors. It has also been shown that free energy minimisation extends the type of probabilistic models described above to encompass retinal stabilisation and oculomotor reflexes (Friston et al. 2010b). A crucial difference here is that we have explicitly considered the problem of dynamics and delays. Our goal was to understand how the system could provide an optimal solution, when it knows (or can infer) the delay between sensing input (in the past) and processing information that informs action (in the future). This endeavour allowed us to build a model—using simple priors over the dynamics of the hidden causes—that reproduces the sorts of anticipatory behaviour seen empirically.

### Limitations

Clearly, there are many aspects of oculomotor control we have ignored in this theoretical work. Foremost, we have used a limited set of stimuli to validate the model. Pursuit initiation was only simulated using a simple sweep of a dot, while smooth pursuit was studied using a sinusoidal trajectory. However, these types of stimuli are commonly used in the literature, as they best characterise the type of behaviour (following, pursuit) that we have tried to characterise: see Barnes (2008) for a review. We have not attempted to reproduce the oscillations at steady state as in Robinson et al. (1986) or Goldreich et al. (1992), although this may help to optimise the parameters of our model in relation to empirical data. The hemi-sinusoidal stimulus is also a typical stimulus for studying anticipatory responses (Avila et al. 2006; Barnes et al. 2000). Further validations of this model would call on a wider range of stimuli and consider and accumulated wealth of neurophysiological and behavioural data (Tlapale et al. 2010).

In this paper, we have focused on inference under a series of generative models of oculomotor control. We have not considered how these models are acquired or learned. In brief, the acquisition of generative models and their subsequent optimisation in terms of their parameters (i.e. synaptic connection strengths) is an important, if distinct, issue. In the context of active inference, model acquisition and perceptual learning can be cast in terms of model selection and parameter optimisation through the minimisation of free energy. Under certain simplifying assumptions, this learning reduces to associative plasticity. A discussion of these and related issues can be found in Friston (2008).

The generative model used in this paper has no explicit representation of space but only the uncertain, vectorial position of a target. We have previously studied the role of prediction in solving problems that are associated with the detection of motion using a dynamical and probabilistic model of spatial integration (Perrinet and Masson 2012). Both that model and the current model entertain a similar problem: that of the integration of local information into a global percept, in both the temporal (this manuscript) and spatial (Perrinet and Masson 2012) domains. We have considered integrating sensory information in the spatial domain: terms of the prediction of sensory causes and their sampling by saccades (Friston et al. 2012b), and of the effects on smooth pursuit of reducing the precision. This manipulation can account for several abnormalities of smooth pursuit eye movements typical of schizophrenia (Adams et al. 2012). In this paper, we have limited ourselves to integrating information over time. It would be nice, in the future, to consider temporal and spatial integration simultaneously.

A final limitation of our model is the simplified modelling of the physical properties of the oculomotor system—due to the biophysics of the eyes and photoreceptors, sensory input contains motion streaks that can influence the detection of motion (Barlow and Olshausen 2004). Furthermore, we have ignored delays in neuronal message passing among and within different levels of the hierarchy: for a review of quantitative data from monkeys, see Salin and Bullier (1995). Finally, we have not considered in any depth the finer details of how predictive coding or Bayesian filtering might be implemented neuronally. It should be noted that predictive coding in the cortex was attended by some early controversies; for example, paradoxical increases in visual evoked responses were observed when prediction error should be minimal. For example, a match between sensory signals and descending predictions can lead to the enhancement of neuronal firing (Roelfsema et al. 1998). The neuronal implementation assumed in our work (see 2) finesses many of these issues. In this (hypothetical) scheme, predictions and prediction errors are encoded by the neuronal activity of deep and superficial pyramidal cells, respectively (Mumford 1992; Bastos et al. 2012). In this scheme, the enhancement of evoked responses is generally thought to reflect attentional gain, which corresponds to the optimisation of the expected precision (inverse variance) of prediction errors, via synaptic gain control  (Feldman and Friston 2010). Put simply, attention increases the gain of salient or precise prediction errors that the predictions are trying to suppress. Indeed, the orthogonal effects of expectations and attention in predictive coding have been established empirically using fMRI (Kok et al. 2011). See Bastos et al. (2012) for a review of the anatomical and electrophysiological evidence that is consistent with the scheme used here.

### Perspectives

Notwithstanding the limitations above, this approach may provide some interesting perspectives on neural computations in the oculomotor system. First, the model presented here can be compared to existing models of the oculomotor system. In particular, any commonalities of function suggest that extant neuromimetic models may be plausibly implemented using a generic predictive coding architecture. Second, the Bayes optimal control solution rests on a computational (anatomical) architecture that can be informed by electrophysiological or psychophysical studies. For example, we have considered only delays at the motor and sensory level. However, delays in axonal conduction between hierarchical levels—within the visual–oculomotor system—may have implications for intrinsic and extrinsic connectivity: in visual search, predictions generated in higher areas (say supplementary and frontal eye fields) may exploit a shorter path, by stimulating the actuator to sample more information (by making an eye movement) rather than accumulating evidence by explaining away prediction errors in lower (striate and extrastriate) cortical levels (Masson et al. 2010). By studying the structure of connections implied by theoretical considerations (see Fig. 3), our modelling approach could provide a formal framework to test these sorts of hypotheses. A complementary approach would be to apply dynamic causal modelling (Friston et al. 2003) to electrophysiological data, using predictive coding architectures, such that transmission delays (and their compensation or modelling) among levels of the visual–oculomotor system could be evaluated empirically. A recent example of using dynamic causal modelling to test hypotheses based upon predictive coding architectures can be found in Brown and Friston (2012). This example focuses on attentional gain control in visual hierarchies.

Second, this work may provide a new perspective for experiments, in particular for the generation of stimuli. We have previously considered such a line of research by designing naturalistic, texture-like pseudo-random visual stimuli to characterise spatial integration during visual motion detection (Leon et al. 2012). We were able to show that the oculomotor system exhibits an increased following gain, when stimuli have a broad spatial frequency bandwidth. Interestingly, the velocities of these stimuli were harder to discriminate relative to narrow bandwidth stimuli—in a two alternative forced-choice psychophysical task (Simoncini et al. 2012). In this work, the authors used competitive dynamics based on divisive normalisation. Moreover, textured stimuli were based on a simple forward model of motion detection (Leon et al. 2012). This may call for the use of more complex generative models to generate such textures. In addition, the use of gaze contingent eye-tracking systems allows real-time manipulation of the configuration (position, velocity, delays) of the stimulus, with respect to eye position and motion. By targeting different sources of uncertainty, at the different levels of the hierarchical model, one might be able to get a better characterisation of the oculomotor system.

The confounding influence of delays inherent in neuronal processing is a strong biophysical constraint on neuronal dynamics. Representations in generalised coordinates of motion provide a potential resolution that may have enjoyed positive evolutionary pressure. However, it remains unclear how neural information, represented in a distributed manner across the nervous system, is integrated with exteroceptive, operational time. The “binding” of different information, without a central clock, seems essential, but the correlate of such a temporal representation of sensory information (independent of delays) has never been observed explicitly in the nervous system. Elucidating the neural representation of temporal information would greatly enhance our understanding of both neural computations themselves and our interpretation of measured electromagnetic (EEG and MEG) signals that are tightly coupled to those computations.

## Appendix

### Appendix 1: Variational free energy

Here, we derive various formations of free energy and show they relate to each other. We start with the quantity we want to bound and implicitly minimise—namely, surprise or the negative log-evidence associated with sensory states $\tilde{s}(t)$ that have been caused by some unknown quantities $\Psi (t)$. These hidden causes correspond to the (generalised) motion (that is position, velocity, acceleration, etc.) of a target that the oculomotor system is tracking.

11 $$\begin{aligned} - \ln p(\tilde{s}) = - \ln \int p(\tilde{s} , \Psi ) d \Psi \end{aligned}$$

We now simply add a non-negative cross-entropy or divergence between some arbitrary (conditional) density $q(\Psi )=q(\Psi | \tilde{\mu })$ and the posterior density $p(\Psi | \tilde{s})$ to create a free energy bound on surprise

12 $$\begin{aligned} F&= - \ln p(\tilde{s}) + \int q(\Psi ) \ln \frac{ q(\Psi )}{ p(\Psi | \tilde{s})} d \Psi \nonumber \\&= - \ln p(\tilde{s}) + D( q(\Psi ) || p(\Psi | \tilde{s}) ) \end{aligned}$$

The cross-entropy term is non-negative by Gibb’s inequality. Because surprise depends only on sensory states, we can bring it inside the integral and use $p(\tilde{s} , \Psi ) = p(\Psi | \tilde{s}) p(\tilde{s})$ to show free energy is a Gibb’s energy $G=- \ln p(\tilde{s} , \Psi )$ expected under the conditional density minus the entropy of the conditional density

13 $$\begin{aligned} F&= \int q(\Psi ) \ln \frac{ q(\Psi )}{ p(\Psi | \tilde{s}) p(\tilde{s})} d \Psi \nonumber \\&= \int q(\Psi ) \ln \frac{ q(\Psi )}{ p(\Psi , \tilde{s})} d \Psi \nonumber \\&= -\int q(\Psi ) \ln p(\Psi , \tilde{s}) d \Psi + \int q(\Psi ) \ln q(\Psi ) d \Psi \end{aligned}$$

This is a useful formulation because it can be evaluated in a relatively straightforward way given a probabilistic generative model $p(\tilde{s} , \Psi )$. A final rearrangement, using $p(\tilde{s} , \Psi ) = p(\tilde{s} | \Psi ) p(\Psi )$, shows free energy is also complexity minus accuracy, where complexity is the divergence between the recognition density $q(\Psi )$ and the prior density $p(\Psi )$

14 $$\begin{aligned} F&= \int q(\Psi ) \ln \frac{ q(\Psi )}{ p(\Psi | \tilde{s}) p(\tilde{s})} d \Psi \nonumber \\&= -\int q(\Psi ) \ln p(\tilde{s} | \Psi ) d \Psi + D( q(\Psi ) || p(\Psi ) ) \end{aligned}$$

### Appendix 2: The maximum entropy principle and the Laplace assumption

If we admit an encoding of the conditional density up to second order moments, then the maximum entropy principle (Jaynes 1957), implicit in the definition of free energy above, requires $q(\Psi | \tilde{\mu }) = \fancyscript{N}(\tilde{\mu }, \Sigma )$ to be Gaussian. This is because a Gaussian density has the maximum entropy of all forms that can be specified with two moments. Assuming a Gaussian form is known as the Laplace assumption and enables us to express the entropy of the conditional density in terms of its first moment or expectation. This follows because we can minimise free energy with respect to the conditional covariance as follows:

15 $$\begin{aligned}&F = G(\tilde{s}, \tilde{\mu }) + \frac{1}{2} tr( \Sigma \partial _{\tilde{\mu } \tilde{\mu }} G ) - \frac{1}{2} \ln | \Sigma | \nonumber \\&G = - \ln p(\tilde{s} , \Psi ) \nonumber \\&\partial _\Sigma F = \frac{1}{2} \partial _{\tilde{\mu } \tilde{\mu }} G - \frac{1}{2} \Pi \end{aligned}$$

so that $\partial _\Sigma F = 0$ implies

16 $$\begin{aligned} \Pi&= \partial _{\tilde{\mu } \tilde{\mu }} G \nonumber \\ F&= G(\tilde{s}, \tilde{\mu }) + \frac{1}{2} \ln | \partial _{\tilde{\mu } \tilde{\mu }} G | \end{aligned}$$

Here, the conditional precision $\Pi (\tilde{s}, \tilde{\mu })$ is the inverse of the conditional covariance $\Sigma (\tilde{s}, \tilde{\mu })$. In short, free energy is a function of generalised conditional expectations and sensory states.

### Appendix 3: Integrating or solving active inference schemes using generalised descents.

Given a generative model or its associated Gibbs energy function, one can now simulate active inference by solving the following set of ordinary differential equations for a system that includes generalised real-world states and internal states of the agent mediating (delayed) action and perception:

17 $$\begin{aligned} \dot{u} = \left[ \begin{array}{c} \dot{\tilde{\varvec{s}}} \\ \dot{\tilde{\varvec{x}}} \\ \dot{\tilde{\varvec{\nu }}} \\ \dot{\varvec{\tilde{\omega }_\nu }} \\ \dot{\varvec{\tilde{\omega }_x}} \\ \dot{\tilde{\mu }} \\ \dot{\tilde{\eta }}\\ \dot{\varvec{a}} \end{array}\right] = \left[ \begin{array}{c} \fancyscript{D}\tilde{\varvec{g}}(\tilde{\varvec{x}}, \tilde{\varvec{\nu }}, \tilde{\varvec{a}}) + \fancyscript{D}\varvec{\tilde{\omega }_\nu } \\ \tilde{\varvec{f}}(\tilde{\varvec{x}}, \tilde{\varvec{\nu }}, \tilde{\varvec{a}}) + \varvec{\tilde{\omega }_x} \\ \fancyscript{D}\tilde{\varvec{\nu }} \\ \fancyscript{D}\varvec{\tilde{\omega }_\nu } \\ \fancyscript{D}\varvec{\tilde{\omega }_x} \\ \fancyscript{D}\tilde{\mu } - \partial _{\tilde{\mu }} F( T(\tau _s -\varvec{\tau _s}) \varvec{\tilde{s}}, \tilde{\mu })\\ \fancyscript{D}\tilde{\eta }\\ - \partial _{a} F( T(\tau _s -\varvec{\tau _s} + \tau _a -\varvec{\tau _a}) \varvec{\tilde{s}}, T(\tau _a -\varvec{\tau _a}) \tilde{\mu }) \end{array}\right] \nonumber \\ \end{aligned}$$

Generalised action $\varvec{\tilde{a}}(t)$ is approximated using discrete values of $\varvec{a}(t)$ from the past. Note that we have included a prior expectation $\tilde{\eta }(t)$ of hidden causes to complete the agent’s generative model of its world. Integrating or solving equation 17 corresponds to simulating active inference. The updates of the collective states over time steps of $\Delta t$ use a local linearisation scheme (Ozaki 1992):

18 $$\begin{aligned} \Delta u&= (\exp (\Delta t \cdot \partial _u \dot{u}) - I)(\partial _u \dot{u})^{-1} \nonumber \\ \partial _u \dot{u}&= \left[ \begin{array}{c@{\quad }c@{\quad }c@{\quad }c@{\quad }c@{\quad }c@{\quad }c@{\quad }c} 0 &{} \fancyscript{D}\partial _{\tilde{\varvec{x}}} \tilde{\varvec{g}} &{} \fancyscript{D}\partial _{\tilde{\varvec{\nu }}} \tilde{\varvec{g}} &{} \fancyscript{D} &{} \ldots &{} &{} &{} \fancyscript{D}\partial _a\tilde{\varvec{g}} \\ &{} \partial _{\tilde{\varvec{x}}} \tilde{\varvec{f}} &{} \partial _{\tilde{\varvec{\nu }}} \tilde{\varvec{f}} &{} &{} I &{} &{} &{} \partial _a\tilde{\varvec{f}} \\ \vdots &{} &{} \fancyscript{D} &{} &{} \vdots &{} \vdots &{} &{} \\ &{} &{} &{} \fancyscript{D}&{} &{} &{} &{} \\ &{} &{} \ldots &{} &{} \fancyscript{D} &{} &{} \ldots &{} \\ - \partial _{\tilde{\mu }\tilde{s}} F &{} &{} \ldots &{} &{} &{} -\fancyscript{D}\partial _{\tilde{\mu }\tilde{\mu }} F &{} - \partial _{\tilde{\mu }\tilde{\eta }} F &{} -\partial _{\tilde{\mu }a} F \\ &{} &{} &{} &{} &{} -\partial _{\tilde{\eta }\tilde{\mu }} F &{} \fancyscript{D} &{} \\ - \partial _{a\tilde{s}} F &{} &{} &{} &{} &{} - \partial _{a\tilde{\mu }} F &{} - \partial _{a\tilde{\eta }} F &{} - \partial _{aa} F \end{array}\right] \nonumber \\ \end{aligned}$$

Details about how to compute the gradients and curvatures pertaining to the conditional expectations can be found in Friston et al. (2010a). These are generally cast in terms of prediction errors using straightforward linear algebra. Because action can only affect free energy through the sensory states, its dynamics are prescribed by the following gradients and curvatures (ignoring higher-order terms):

19 $$\begin{aligned}&\partial _a F = (\partial _a \tilde{\varepsilon }^{(1)}_\nu ) \cdot \Pi ^{(1)}_a T(\tau _a -\varvec{\tau }_a) \tilde{\varepsilon }^{(1)}_\nu \nonumber \\&\partial _{aa} F = (\partial _a \tilde{\varepsilon }^{(1)}_\nu ) \cdot \Pi ^{(1)}_a T(\tau _a -\varvec{\tau }_a) (\partial _a \tilde{\varepsilon }^{(1)}_\nu ) \nonumber \\&\partial _a \tilde{\varepsilon }^{(1)}_\nu = T(\tau _s-\varvec{\tau }_s) \partial _a \varvec{\tilde{s}}(t) \nonumber \\&\partial _a \varvec{\tilde{s}} = \partial _a \varvec{\tilde{g}} + \partial _{\tilde{\varvec{x}}} \varvec{\tilde{g}}\left( \sum _i \fancyscript{D}^{-i}(\partial _{\tilde{x}} \tilde{\varvec{f}}) ^{i-1}\right) \partial _{a} \tilde{\varvec{f}} \end{aligned}$$

The partial derivative of the sensory states with respect to action and is specified by the generative process. In biologically plausible instances of this scheme, this derivative would have to be computed on the basis of a mapping from action to sensory consequences. It is generally assumed that agents are equipped with $\partial _a \varvec{\tilde{s}}$ epigenetically, because it has a simple form. For example, contracting a muscle fibre elicits a proprioceptive stretch signal in a one-to-one fashion. The precision matrix $\Pi ^{(1)}_a$ in Eq. 19 is specified such that only proprioceptive prediction errors with these simple forms have nonzero precision. This can be regarded as the motor gain in response to proprioceptive prediction errors. Equation 18 may look complicated but can be evaluated automatically using numerical derivatives for any given generative model. All the simulations in this paper used just one routine—$\mathsf {toolbox/DEM/spm\_ADEM.m}$. All figures are reproducible and summarised in the script $\mathsf {toolbox/DEM/ADEM\_oculomotor\_delays.m}$. Both are available as part of the SPM software (http://www.fil.ion.ion.ucl.ac.uk/spm).

## Footnotes

1 We have a made a slight approximation here because $T(\tau _a) \tilde{\mu }(t-\varvec{\tau }_a) = T(\tau _a-\varvec{\tau }_a) \tilde{\mu }(t)$ when, and only when, the free energy gradients are zero and $\dot{\tilde{\mu }}(t) = \fancyscript{D} \tilde{\mu }(t)$. Under the assumption that the perceptual destruction of these gradients is fast, in relation to action, this can be regarded as an adiabatic approximation.
